# Discovery of new diketopiperazines inhibiting *Burkholderia cenocepacia* quorum sensing *in vitro* and *in vivo*

**DOI:** 10.1038/srep32487

**Published:** 2016-09-01

**Authors:** Viola C. Scoffone, Laurent R. Chiarelli, Vadim Makarov, Gilles Brackman, Aygun Israyilova, Alberto Azzalin, Federico Forneris, Olga Riabova, Svetlana Savina, Tom Coenye, Giovanna Riccardi, Silvia Buroni

**Affiliations:** 1 Dipartimento di Biologia e Biotecnologie, Università degli Studi di Pavia, Via Ferrata, 1-27100 Pavia, Italy; 2 Lab for Biomedicinal Chemistry, Bach Institute of Biochemistry, Research Center of Biotechnology of the Russian Academy of Sciences, Moscow 119071, Russia; 3 Lab of Pharmaceutical Microbiology, Ghent University, Ottergemsesteenweg 460, Ghent, 9000, Belgium; 4 Department of Microbiology, Baku State University, Z. Khalilov 23, AZ1148, Baku, Azerbaijan; 5 Neurosurgery, Dipartimento di Scienze Clinico-Chirurgiche e Pediatriche, Università degli Studi di Pavia, Fondazione IRCCS Policlinico S. Matteo, Pavia, Italy; 6 IGM-CNR, Via Abbiategrasso 207, 27100, Pavia, Italy

## Abstract

*Burkholderia cenocepacia*, an opportunistic respiratory pathogen particularly relevant for cystic fibrosis patients, is difficult to eradicate due to its high level of resistance to most clinically relevant antimicrobials. Consequently, the discovery of new antimicrobials as well as molecules capable of inhibiting its virulence is mandatory. In this regard quorum sensing (QS) represents a good target for anti-virulence therapies, as it has been linked to biofilm formation and is important for the production of several virulence factors, including proteases and siderophores. Here, we report the discovery of new diketopiperazine inhibitors of the *B. cenocepacia* acyl homoserine lactone synthase CepI, and report their anti-virulence properties. Out of ten different compounds assayed against recombinant CepI, four were effective inhibitors, with IC_50_ values in the micromolar range. The best compounds interfered with protease and siderophore production, as well as with biofilm formation, and showed good *in vivo* activity in a *Caenorhabditis elegans* infection model. These molecules were also tested in human cells and showed very low toxicity. Therefore, they could be considered for *in vivo* combined treatments with established or novel antimicrobials, to improve the current therapeutic strategies against *B*. *cenocepacia*.

Cystic Fibrosis (CF) is an autosomal recessive disease affecting approximately 100,000 people worldwide, and is considered a rare disease^1^. The genetic defect causes the malfunction of the chloride channel termed Cystic Fibrosis Transmembrane conductance Regulator (CFTR), which leads to formation and build-up of sticky mucus. This results in impairment of mucociliary clearance of opportunistic pathogens, making the control of infections a major concern^2^,^3^.

*Burkholderia cenocepacia*, a member of the *Burkholderia cepacia* complex (Bcc), is one of these opportunistic pathogens. Infection with *B. cenocepacia* is often associated with poor clinical outcome and high mortality resulting from a decline of lung function leading to fatal pneumonia^4^,^5^. These bacteria are intrinsically multidrug resistant and can form biofilms in the airways, thus increasing their tolerance to high concentrations of drugs^6^,^7^. Consequently, innovative solutions are needed to improve the effectiveness of current antibacterial therapies.

Quorum sensing (QS) is an intercellular cell density dependent communication process, based on the synthesis and secretion of signal molecules^8^. These molecules are sensed by bacteria through specific receptors, which in turn mediate the induction and/or the repression of target genes in relation to the signal molecule concentration. The involvement of QS in biofilm formation and expression of other major virulence factors such as proteases, siderophores, and toxins is well-established^9^,^10^, and the *Burkholderia* QS system is an interesting candidate drug target^11^,^12^.

The hypothesis is that interfering with the activity of signal molecule synthases renders the bacteria unable to produce virulence factors and thus less able to colonize the host. Furthermore, therapies directed at inhibiting QS (as well as other anti-virulence treatments) do not directly kill the bacteria, making the development of drug resistance less likely. Finally, these QS anti-virulence drugs might be used in combination with established or novel antimicrobials so as to improve the currently available therapies^11^.

All *Burkholderia* species encode at least one QS system consisting of an Acyl Homoserine Lactone (AHL) synthase and an AHL receptor^13^. *B. cenocepacia* J2315 possesses two complete AHL QS systems (CepIR and CciIR) and one orphan (a gene encoding a regulator not paired up with a synthase, CepR2) plus the *Burkholderia* Diffusible Signal Factor (BDSF)-based system, RpfF_BC_^14^,^15^,^16^.

CepI is responsible for the synthesis of N-octanoyl-homoserine lactone (C8-HSL) and, in smaller amounts, of N-hexanoyl-homoserine lactone (C6-HSL) starting from acylated acyl-carrier protein (acyl-ACP) and S-adenosyl methionine (SAM) (Fig. 1)^17^.

In a previous study, the properties of various *B. cenocepacia* mutants affected in QS were characterized^18^. Our results confirmed the involvement of CepI in biofilm formation, protease production and virulence. Moreover, those results highlighted the interplay among the AHL and BDSF-based systems, suggesting that the BDSF system controls the AHL-based QS system^18^.

In the present study, CepI from *B. cenocepacia* J2315 was characterized by using structural bioinformatics and by heterologous production and purification in *Escherichia coli*. An enzymatic assay was set up (based on previous work^19^) and was shown to be suitable for high-throughput screening of compounds targeting CepI.

To this end, ten new molecules were synthesized based on the diketopiperazines scaffold (diketopiperazines were previously described as QS inhibitors^20^). The molecules were tested for inhibitory activity against CepI. Four of these compounds interfered with CepI activity in a dose-dependent manner. Further testing highlighted that these inhibitors also impaired the ability of *B. cenocepacia* J2315 to produce proteases, siderophores, and to form biofilms *in vitro*. Moreover, the administration of the compounds increased survival of *Caenorhabditis elegans* nematodes infected with *B. cenocepacia* J2315, suggesting that the virulence of the strain was also attenuated under *in vivo* conditions.

## Results

### CepI enzymatic activity is inhibited by four diketopiperazine derivatives

**Figure 2:**
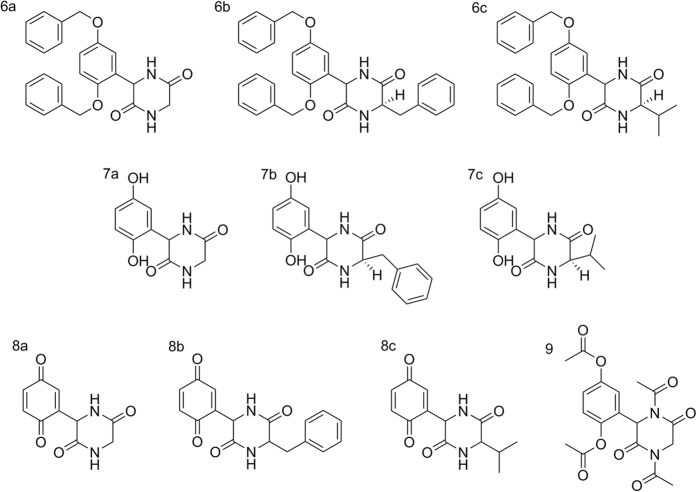
Chemical structure of diketopiperazines investigated in this work.

In order to identify molecules able to inhibit the QS enzyme CepI, the recombinant protein was purified (Supplementary Fig. S1), characterized (Supplementary Fig. S2) and used to test a series of new compounds (Fig. 2). The purified recombinant CepI was catalytically active, showing steady state kinetic parameters towards C8-ACP very similar to those of other AHL synthases^19^,^21^ (Supplementary Fig. S2). Moreover, the protein was rather stable in 50 mM TrisHCl pH 8.0, 150 mM NaCl, 1 mM DTT, 10% glycerol and it may be stored in these conditions at −80 °C for over 1 year without significant loss of activity. Then the sample can be kept at 4 °C for maximum 1 week (data not shown). Initially, the efficacy of compound 1 from Christensen *et al*. (3-(4-methylpiperazin-1-yl)(pyridin-2-yl)methyl]-2-phenyl-1H-indol-1-ol) was tested, as it has been demonstrated to be very active against the *Burkholderia mallei* AHL-synthase^19^. The compound was also found to be effective against the *B. cenocepacia* CepI (IC_50_: 7.2±0.2 μM), thus confirming that the recombinant enzyme is suitable for inhibitor screening.

**Figure 3:**
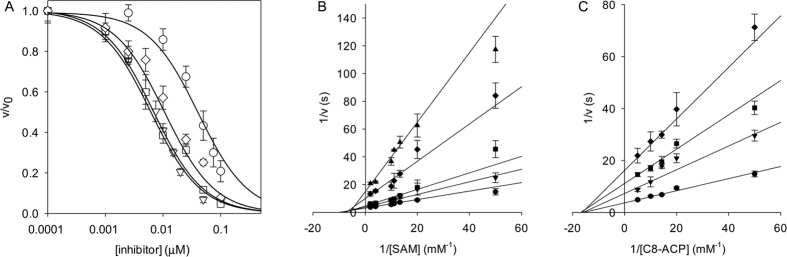
Inhibition of CepI activity. (**A**) IC_50_ determination of **6a** (○), **8a**(⋄), **8b** (▿) and **8c** (◽) against CepI. IC_50_ values were determined by fitting the experimental data, as reported in Materials and Methods. (**B**) Reciprocal plot of the steady state kinetic analysis towards SAM of CepI, in the presence of different concentrations of **8b** (⦁ 0 mM; ▾ 0.01 mM; ◾ 0.02 mM; ♦ 0.05 mM; ▴ 0.1 mM). **(C)** Reciprocal plot of the steady state kinetic analysis towards C8-ACP of CepI in the presence of different concentrations of **8b** (⦁ 0 mM; ▾ 0.01 mM; ◾ 0.02 mM; ♦ 0.05 mM).

For this purpose, ten novel diketopiperazines were synthetized (Fig. 2) based on the structure of diketopiperazines which act as QS inhibitors^20^. A redox moiety was introduced in the molecule using the quinone function and the corresponding quinones and hydroquinones were synthesized. Four of these newly synthesized compounds (**8a**, **8b**, **8c**, and to a lesser extent **6a**), were effective inhibitors of the CepI enzymatic activity, with IC_50_ values ranging from 5 to 30 μM (Supplementary Table S1, Fig. 3A).

To better characterize the mechanism of action of this newly synthesized class of CepI inhibitors, the apparent inhibition constants of the most active compound (**8b**) were determined. The compound behaved as a non-competitive inhibitor towards both C8-ACP and SAM substrates, showing an apparent *K*_i_ value of 17±0.6 μM (Fig. 3B,C), proving to be very active in inhibiting CepI *in vitro*.

In order to determine structure-activity relationship of the inhibitors, we wanted to determine the structure of CepI. However, despite numerous trials, the enzyme proved to be recalcitrant to crystallization. Therefore, we performed extensive structural bioinformatics predictions through comparative evaluation of multiple structural prediction and docking algorithms, as described in the Supplementary Materials and Methods. The high sequence conservation among homologous AHL synthases of known three-dimensional structure such as TofI^22^, EsaI^23^, LasI^24^ and CepI (Supplementary Table S2, Supplementary Fig. S3), as well as the broad structural fold conservation with other prokaryotic and eukaryotic acyltransferase homologs allowed clear identification of the amino acid residues involved in substrate recognition and catalysis (Supplementary Fig. S4). Overall, CepI shares the structural architecture of homologous AHL synthases^25^, characterized by a broad, V-shaped substrate binding pocket that crosses the entire enzyme fold and shows very high sequence conservation (Supplementary Fig. S4). Previously reported mutagenesis studies on CepI homologs identified the critical amino acid residues implicated in synthase enzymatic activity^25^,^26^,^27^. Sequence alignments and comparison of our CepI homology model with available crystal structures shows complete conservation of all residues essential for enzymatic activity (Supplementary Fig. S3). Residues Phe27, Trp33 and Arg104 are likely implicated in stabilization of the both substrates^23^,^25^, whereas residues Asp45, Glu101 and Ser103, fully conserved in all AHL synthases, have been implicated in catalysis^22^,^23^,^25^. A broad region spanning from residue 90 to 180 shapes a shallow hydrophobic tunnel, likely responsible for hosting the acyl moiety of the C8-ACP substrate during catalysis. Of note, all residues lining this putative acyl-binding pocket are conserved between CepI and TofI (Supplementary Fig. S3); both CepI and TofI synthesize C8-AHL. The C-terminus is characterized by the highest sequence heterogeneity among homologous enzymes. Nevertheless, multiple solvent-exposed positively charged residues, including Arg150, Arg154, and Arg161, implicated in the recognition of the negatively charged C-terminus of the ACP carrier and therefore essential for productive substrate binding^24^, are conserved (Supplementary Fig. S3).

Attempts to identify the putative binding site of compound **8b** to the CepI structural model using molecular docking consistently showed the presence of multiple high-affinity contact sites spread around the CepI structure (Supplementary Fig. S5). Interestingly, all putative interaction sites identified by docking were outside of the SAM and acyl substrate binding site, supporting the biochemical evidence for non-competitive inhibition. Among these, putative binding sites with predicted strong stabilization energies were found proximate to the loop covering residues 33–47, critical for SAM stabilization in TofI^22^, and near the Arg residues involved in interaction with ACP (Supplementary Fig. S5).

### Quorum sensing inhibitor (QSI) MIC in planktonic cells

1 to 128 μg/ml of the compounds **6a**, **6b**, **6c**, **7a**, **7b**, **7c**, **8a**, **8b**, **8c**, and **9** were added to planktonic *B. cenocepacia* J2315 cells. *B. cenocepacia* was able to grow even at the highest concentration tested, demonstrating that the compounds did not show any bactericidal or bacteriostatic activity (Supplementary Table S1, Supplementary Methods).

To determine synergy, 20 μg/ml of **6a** or **8b** was combined with two-fold dilutions of ampicillin, aztreonam, ceftazidime, chloramphenicol, ciprofloxacin, gentamicin, kanamycin, levofloxacin, meropenem, nalidixic acid, norfloxacin, sparfloxacin, streptomycin, tetracycline, tobramycin, or trimethoprim (See Supplementary Methods).

Neither compound altered the susceptibility of *B. cenocepacia* toward the antibiotics tested. In fact, the fractional inhibitory concentration (FIC) indices were >0.5 for all combinations, indicating that no synergistic activity occurred and that the interactions observed were indifferent (data not shown).

### QSIs affect the *in vitro* production of proteases and siderophores in *B. cenocepacia*

**Figure 4:**
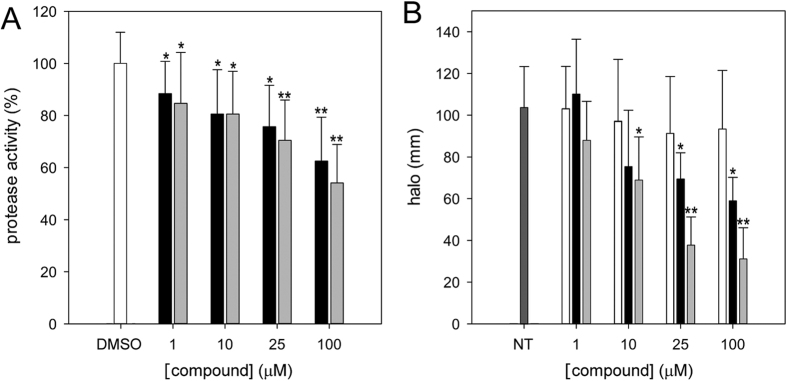
Protease and siderophore production in the presence of QS inhibitors. (**A**) Percentage of protease activity in the presence of compounds **6a** (light grey bars) or **8b** (black bars) compared to the treatment with DMSO (white bar). (**B**) Siderophore production in the presence of DMSO (white bars, p > 0.4), **6a** (light grey bars) and **8b** (black bars) compared to the untreated cells (NT, dark grey bar). *p < 0.05; **p < 0.01.

We subsequently evaluated the effect of **6a** and **8b** on the production of putative virulence factors. It was previously shown that the protease production in *B. cenocepacia* is controlled by AHL-based signaling^28^,^29^. In *B. cenocepacia* J2315 the protease activity decreased significantly (p < 0.05) in the presence of increasing concentrations of the two compounds (1–100 μM) (Fig. 4A). More precisely, compound **8b** induced a dose-dependent decrease in the production of proteases of 12–38% respect to the cells treated with the solvent DMSO, while the compound **6a** led to a decrease of 16–46%.

To assess if siderophore production was influenced by the two inhibitors, four different concentrations of **8b** and **6a** were tested (1, 10, 25 and 100 μM) with cells grown on CAS agar medium in the presence of low iron concentrations. On these plates, siderophores remove iron from the CAS dye complex, resulting in a blue-to-orange color change around the colonies^30^. Both compounds were able to decrease siderophore production in *B. cenocepacia* J2315 by 25–42% and 13–69%, respectively (p < 0.05) (Fig. 4B).

### Effect of QSIs on biofilm formation

In order to assess the effect of compounds **6a** and **8b** on biofilm morphology, we evaluated biofilm formation in the presence or absence of the compounds. While in the untreated controls (i.e. biofilm treated with solvent control) the morphological variation of the biofilm was minor, significant morphological differences were observed for the treated biofilms.

**Figure 5:**
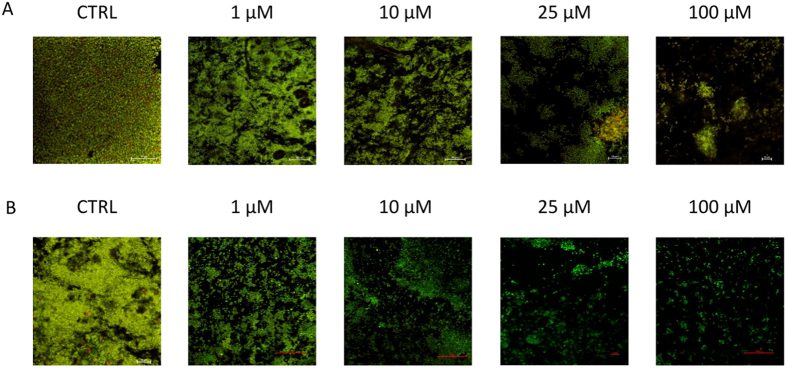
Representative microscopic images of *B. cenocepacia* J2315 biofilms. Biofilms formed in the absence (CTRL, i.e. solvent control) or presence of different concentrations of **6a** (**A**) or **8b** (**B**).

*B. cenocepacia* J2315 biofilms formed in the absence of compounds were able to almost completely cover the surface of the well (Fig. 5). In contrast, biofilms formed in the presence of 10 μM, 25 μM and 100 μM of **6a** and **8b** were less structured, contained fewer cells and were unable to colonize the entire surface of the well (Fig. 5). Furthermore, minor differences were also observed between untreated control biofilm and those treated with 1 μM of **6a** and **8b**, suggesting that **6a** and **8b** affect biofilm formation even at very low concentrations. However, we also observed minor variations in biofilm morphology in the solvent-treated controls, and it remains to be determined whether the minor differences observed at low inhibitor concentrations are biologically relevant.

### Effect of QSIs on survival of *C. elegans* infected by *B. cenocepacia*

*C. elegans* infected with *B. cenocepacia* J2315 was used to evaluate the ability of the compounds to protect against infection^18^,^31^. The compounds themselves did not display any toxic effect against *C. elegans* nematodes, since no significant differences in survival were observed after 24 h and 48 h in uninfected *C. elegans* treated with the compounds in concentrations ranging between 1–25 μM (Table 1). In contrast, survival was significantly decreased when infected *C. elegans* received no treatment (survival of 65% ± 7% and 35% ± 18% after 24 h and 48 h, respectively compared to 96% ± 5% and 95% ± 5% survival for uninfected untreated nematodes) (Table 1). Both **6a** and **8b** significantly protected *C. elegans* nematodes against infection with *B. cenocepacia* J2315 (Table 1). Similar levels of survival were observed after 24 h between uninfected *C. elegans* nematodes and nematodes infected with *B. cenocepacia* J2315 which were treated with 25 μM of **6a** or **8b** (96% ± 5%, 91% ± 9% and 91% ± 5% after 24 h, respectively). In addition, both compounds significantly increased survival of infected nematodes after 24 h and 48 h even when administered concentrations were as low as 1 μM (Table 1).

### Effect of 8b and 6a on HeLa cells

**Figure 6:**
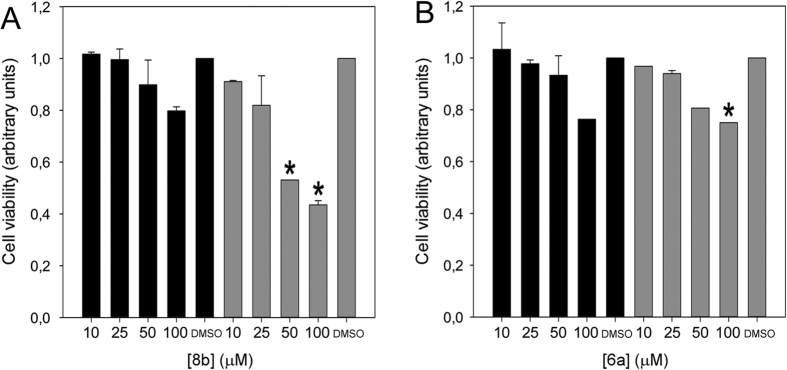
Compound toxicity assessment. HeLa cell viability in the presence of 8b (**A**) and **6a** (**B**), after 24 h (black bars) and 48 h (grey bars) of incubation with different compound concentrations. DMSO, cells treated with the highest amount of solvent. *Indicates a significant decrease in cell viability (p < 0.05).

The toxicity of the newly synthesized compounds was tested on HeLa cells which were exposed to **8b** and **6a** at the concentrations of 10, 25, 50 and 100 μM. Following exposure, cells were counted after 24 h and 48 h. Compound **8b** at 100 μM already caused approximately a 20% reduction in cell viability after 24 h (Fig. 6); the effect was stronger at 48 h. Significant toxicity (p < 0.05) could be observed using 50 μM compound concentration after 48 h (approximately 50% cell viability reduction) (Fig. 6). For both concentrations, microphotographs collected at 72 h confirmed cell death using either 50 and 100 μM concentrations (Supplementary Fig. S6).

Concentrations as low as 25 μM did not induce cell death (Supplementary Fig. S6), however the cells stopped growing between 24 h and 48 h.

On the other hand, compound **6a** displayed a lower cell toxicity (Fig. 6) and significant effects (p < 0.05) on cell growth were observed only after 48 h treatment at a concentration of 100 μM. Furthermore, the fact that **6a** did not induce cell death was obvious from microphotographs after 72 h (Supplementary Fig. S6).

### Discussion and Conclusions

There is a lack of new therapeutic solutions for *B. cenocepacia* infections, and targeting the QS system of this organism could be a promising approach, potentially with less pressure to select resistant strains^32^. This is particularly important as antimicrobial resistance is recognized worldwide as one of the major public health concerns of our century^33^. Different strategies can be pursued to block QS systems: the inhibition of the synthesis of the signal molecule; targeting the signal molecule itself (by degradation or deactivation) and/or antagonizing the regulator^11^.

Several recent studies reported the use of molecules able to inhibit QS regulators, such as thiazolidinedione analogues that block the DNA binding ability of LuxR in *Vibrio harveyi*^34^ or AHLs and their precursor synthesis in *Pseudomonas aeruginosa*^35^. Moreover, other groups developed analogues of autoinducing peptides to inhibit QS receptors and attenuate virulence in *Staphylococcus aureus*^36^ and *Staphylococcus epidermidis*^37^.

In the present study we focused on a third possible target of the QS pathway, the AHL synthase CepI of *B. cenocepacia* J2315. Diketopiperazine molecules inhibit the enzymatic activity of CepI *in vitro*, and by doing so downregulate the production of virulence factors; such hypothesis was clearly confirmed regarding protease production. The production of siderophores is another well-recognized potential virulence factor implicated in the pathogenesis of *B. cenocepacia* infections, as demonstrated in CF clinical isolates^38^. An example would be pyochelin: this molecule plays a role in tissue injury in addition to iron acquisition, as it is an efficient catalyst for hydroxyl radical formation^39^. CepR was shown to negatively regulate its own expression as well as the siderophore ornibactin biosynthesis through the *pvdA* gene^39^. Lewenza and Sokol (2001)^39^ demonstrated that iron increases *cepR* expression, which could lead to higher levels of *pvdA* repression in WT strains, and that *cepR* mutations have a greater effect on *pvdA* expression in the presence of high iron concentrations in the culture medium. Moreover, iron acquisition *via* the siderophore ornibactin (encoded by *pvdA*) was confirmed to play a role in the early stages of *B. cenocepacia* colonization^40^. On the other hand, previous studies demonstrated that the *B. cenocepacia* H111 strain (which lacks the CciIR system) mutated in *cepR*, *cepR2* or both show a diminished production of siderophores^41^. Moreover, in a *B. cenocepacia* J2315 double mutant in *cepI*-*cciI* the ornibactin synthase encoding gene *orbI* was significantly downregulated^18^. Treatment of *B. cenocepacia* J2315 with both compounds **8b** and **6a** resulted in a diminished production of siderophores, thus demonstrating their efficacy against virulence.

Diketopiperazines have been also shown to affect biofilm formation of *B. cenocepacia* J2315. This is in accordance with our previous findings, in which biofilms formed by a mutant lacking the *cepI* gene were much thinner, less densely packed and covered only parts of the surface^18^. Diketopiperazines were also assayed against planktonic growing cells but, as expected for compounds which hit the non-essential QS pathway, they did not show any antimicrobial property, as demonstrated by MIC determination experiments. On the contrary, very promising results were obtained in *C. elegans*, where the effect of two inhibitors was clearly demonstrated *in vivo*, even at low concentrations (down to 1 μM, after 48 h treatments). In a previous study^18^ we constructed mutants in *B. cenocepacia* strain J2315, in which genes encoding CepI (BCAM1870), CciI (BCAM0239a) and the BDSF synthase (BCAM0581) were inactivated (either in single, double or triple mutants) and several phenotypic properties (including biofilm formation and virulence in *C. elegans*) were investigated. Interestingly, although all QS mutant strains displayed an impaired ability to form biofilms, this effect on biofilm formation was most pronounced in mutant strains lacking the *cepI* gene. While the WT strain formed a thick densely packed biofilm, biofilms formed by the *cepI* mutant were much thinner, less densely packed and covered only parts of the surface. Similar observations were made when the *B. cenocepacia* J2315 was treated with the compounds described in this study. In addition, our previous publication indicated that the pathogenicity of *B. cenocepacia* J2315 towards *C. elegans* nematodes was significantly impaired when QS genes were deleted in this strain. In the present study we observed that, in absence of treatment, the survival of infected nematodes was only of 65%, whereas their survival reached maximum values of 91% when they received treatment with 25 μM of **6a** or **8b**, respectively; these levels of survival are similar to those of nematodes infected with a QS-deficient mutant.

Furthermore, the tests performed with HeLa cells confirmed the non-toxicity of diketopiperazines. This last observation is very important, because despite the fact that many QSIs have been described^42^ no candidates have, as of yet, reached clinical trials, either because of their toxicity or due to their lack of *in vivo* activity^43^,^44^.

Despite the lack of structural data on CepI, the high degree of sequence identity with TofI and other AHL homologs allowed generation of a reliable structural model of the enzyme, which enabled evaluation of the structural features critical for the catalytic activity of CepI. Docking of the newly discovered **8b** inhibitor onto the CepI structural model consistently showed multiple candidate binding sites on the enzyme surface, distant from the CepI catalytic site but in regions that may have strong implications in substrate recognition and catalysis. It should be pointed out that, at present, the exact mechanism of action of QSIs is poorly understood^45^. As such the design of our compounds and their screening for inhibition of CepI was based on a high-throughput screening assay. Structural data on CepI or related AHL synthases in complex with these molecules will nevertheless be necessary to understand the molecular mechanism of non-competitive enzyme inhibition.

In conclusion, alternative strategies to the use of antimicrobials are emerging as favourable against bacteria intrinsically resistant to the classical antibiotics, as in the case of *B. cenocepacia*. In this respect, our data show that diketopiperazines are very promising therapeutic candidates.

## Methods

### QSI synthesis

**Figure 7:**
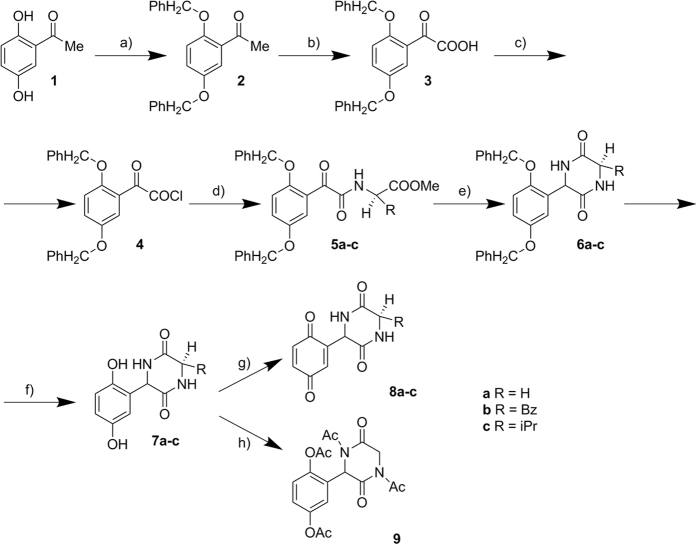
Reagents and conditions. (**a**) PhCH_2_Cl, EtOH, K_2_CO_3_, reflux, 4 h, 77%; (**b**) SeO2, pyridine, 100 °C, 3 h, 81%; **(c)** SOCl_2_, CCl4, DMF, reflux, 3 h; (**d**) H_2_NCHRCOOMe, Et_3_N, DMF, 48 h; (**e**) NH_3_/EtOH, 48 h, 150 °C; (**f**) H_2_, 10% Pd/C, DMF; (**g**) HClO_4_, H_2_SO_4_, HNO_3_, AcOH, 3 h; (**h**) Ac_2_O, reflux, 6 h, 69%.

The general procedure for the synthesis of diketopiperazine derivatives is described in the Supplementary Materials and Methods. Briefly, synthesis of diketopiperazines was performed using 2′,5′-dihydroxyacetophenone **1** as starting material. The first step was the benzyl protection of hydroxyl group. This compound was then oxidized with selenium oxide in pyridine and the resulting acid **3** was turned into the corresponding chloroanhydride **4** by treatment with thionyl chloride in presence of catalytic quantities of DMF. Aminoesters reacted with the chloroanhydride **3** in DMF and in presence of triethylamine with formation of the corresponding diphenoxyderivatives **5a-c**. Their cyclisation to the diketopiperazines **6a-c** was made in bomb in presence of excess of ammonia. De-protection of hydroxygroups was performed by hydrogenation in the presence of palladium of carbon, with high yield, and oxidation of the product followed to generate quinine derivatives **8a-c**. Hydroquinone **7a** was also refluxed with acetic acid anhydride to result in the formation of the corresponding 4 acetyl derivative 9 (Fig. 7).

### CepI heterologous expression and purification

*cepI* was amplified using CepISUMOfor (5′-ATGCAGACCTTCGTTCAC-3′) and CepISUMOrev (5′-TCAGGCGGCGATAGCTTG-3′) as primers and *B. cenocepacia* J2315 genomic DNA as template. Cycling conditions were 4 min 94 °C, 30 cycles 30 s 98 °C, 30s 50 °C, 1 min 72 °C and 10 min 72 °C. The amplified fragment was cloned into pETSUMO (Invitrogen). For protein expression, *E. coli* BL21(DE3) cells were transformed with the pET-SUMO-*cepI* plasmid and grown at 37 °C until an OD_600nm_ of 0.6–0.8 was reached. Recombinant expression was induced with 0.5 mM isopropyl-β-thiogalactopyranoside (IPTG), at 25 °C O/N. Cells were harvested by centrifugation, resuspended in 50 mM TrisHCl pH 8.0, 300 mM NaCl, 5% glycerol, 1 mM DTT (Buffer A), plus 5 mM imidazole, 1:100 Protease Inhibitor Cocktail (Sigma-Aldrich), 50 μg/ml DNase and disrupted by sonication. The cell-free extract, obtained after 50 min centrifugation at 30,000 × *g*, was applied onto an HisTrap column (1 ml, GE-Healthcare), washed with 20 mM imidazole and the protein was eluted with 350 mM imidazole. Tag cleavage was achieved by O/N incubation with 0.3 mg of SUMO protease dialyzed against Buffer A, followed by a second purification with the HisTrap column to remove both the cleaved tag and the SUMO protease. The protein was finally concentrated and loaded onto a HiLoad 26/60 Superdex 200 size exclusion chromatography column (GE Healthcare), equilibrated in 50 mM TrisHCl pH 8.0, 150 mM NaCl, 1 mM DTT. Protein quality was checked by SDS-PAGE and its concentration was evaluated by measuring its absorbance at 280 nm (ε = 26720 M^−1^ cm^−1^).

### C8-ACP preparation

Octanoyl-ACP (C8-ACP), was prepared as previously reported^46^,^47^, using the *Bacillus subtilis* phosphopantetheinyl transferase (Sfp) and the *E. coli* acyl carrier protein (ACP), both obtained in recombinant form (see Supplementary Information).

1 mM ACP was incubated with 10 μM Sfp, 10 mM octanoyl-CoA, 10 μM MgCl_2_, 1 mM DTT in 100 mM TrisHCl pH 8.0, at 37 °C for 16 h. Sfp was precipitated with 75% saturation (NH_4_)_2_SO_4_ for 1 h at 4 °C, and removed by centrifugation. ACP was precipitated O/N at −20 °C with two volumes of acetone. The obtained C8-ACP was collected by centrifugation, dried, suspended in 25 mM TrisHCl pH 7.5, and stored at −80 °C until use. The conversion of ACP was checked by conformation sensitive native PAGE (20% polyacrylamide and 0.5 M urea)^48^.

### Enzymatic assay

Determination of the CepI enzyme activity was performed spectrophotometrically by measuring *holo*-ACP formation with dichlorophenylindophenol (DCPIP; ε = 19100 M^−1^ cm^−1^) at 37 °C according to Christensen *et al*.^19^. Reaction mixtures typically contained 50 mM 4-(2-hydroxyethyl)-1-piperazineethanesulfonic acid pH 7.5 (HEPES), 0.005% Nonidet P-40, 0.13 mM DCPIP, 70 μM C8-ACP, 40 μM S-adenosyl methionine (SAM), 4 μM CepI and the reaction was started by addition of SAM after pre-incubation for 10 min.

Steady-state kinetic parameters *K*_m_ and *k*_cat_ were determined by assaying the enzymes in triplicate at 8 different substrate concentrations, and fitting the data to the Michaelis-Menten equation using Origin 8 software.

CepI inhibition was initially screened for all compounds at 100 μM (dissolved in DMSO). For compounds that significantly inhibited the enzyme activity in these conditions, IC_50_ and *K*_i_ values were determined. To determine IC_50_, the enzyme activities were measured in presence of different concentrations of compounds and values determined with equation (1):


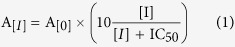


where A_[I]_ is the enzyme activity at inhibitor concentration [I] and A_[0]_ is the enzyme activity without inhibitor.

*K*_i_ determination was determined using the equation (2) for noncompetitive inhibition^49^:


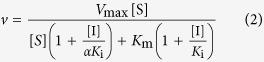


### Protease assay

The protease activity assay was conducted using azocasein, as previously described^18^. Briefly, *B. cenocepacia* J2315 was grown O/N in LB at 37 °C in the presence of increasing concentrations (1–100 μM) of CepI inhibitors **6a** or **8b**. In order to keep the same amount of solvent added, different stock solutions of the inhibitors were prepared. The corresponding amount of DMSO was used as control. The OD at 600 nm was determined and 250 μl of cell-free supernatant were incubated with 250 μl azocasein (5 mg/ml in 100 mM TrisHCl pH 8.0) at 37 °C for 1 h. This reaction was blocked by adding 50 μl 10% trichloroacetic acid and samples were centrifuged. The supernatant was transferred to 350 μl NaOH (525 mM) and the optical density at 420 nm was measured.

### Siderophore production assay

Siderophore activity present in the culture was tested using Chromeazurol S (CAS) agar plates^30^. *B. cenocepacia* J2315 was grown O/N in LB at 37 °C in the presence of increasing concentrations (1–100 μM) of the CepI inhibitors **6a** and **8b** and 2 μl of each culture were spotted on CAS agar plates and incubated for 48 h at 37 °C.

### Biofilm assay

*B. cenocepacia* J2315 was grown overnight in Mueller Hinton (MH), centrifuged and resuspended in MH to an OD_590 nm_ of 0.2. Ninety-nine μl of the bacterial suspension were transferred to the wells of black flat-bottom 96-well microtiter plate with a glass bottom (TPP). One μl control solution (i.e. solvent without active molecule) was added to the control wells (untreated) and 1 μl of **6a** or **8b** solution (concentrated 100 times) was added to the test wells (treated). Bacteria were allowed to adhere and grow without agitation for 4 h at 37 °C. After 4 h, plates were emptied and washed with sterile physiological saline (PS). Following this wash step, all wells were filled with 99 μl MH and 1 μl of either the solvent solution or of a solution of **6a** or **8b**, the plate was then incubated for 20 h at 37 °C. After 24 h biofilm formation, medium was removed, biofilms were washed with 100 μl PS and 100 μl of a staining solution (containing 3 μl of SYTO9 and 3 μl of propidium iodide in 1 ml of PS; Life technologies) were added. These plates were incubated in the dark for 15 min at room temperature and the biofilm was visualized with a Nikon C1 confocal laser scanning microscope (Nikon Benelux, Brussels, Belgium) as previously described^50^. Tests were performed on at least three wells for each condition and representative images are shown.

### Virulence assay in *Caenorhabditis elegans*

*C. elegans* N2 (*glp-4; sek-1*) was propagated under standard conditions, synchronized by hypochlorite bleaching, and cultured on nematode growth medium using *E. coli* OP50 as a food source, as described previously^51^,^52^. The *C. elegans* survival assay was carried out as described previously^31^. In brief, synchronized worms (L4 stage) were suspended in a medium containing 95% M9 buffer, 5% brain heart infusion broth (Oxoid), and 10 μg of cholesterol (Sigma-Aldrich) per ml. Fifty μl of this suspension of nematodes were transferred to the wells of a 96-well microtiter plate where 49 μl of medium were added to the uninfected control wells. An overnight bacterial culture was centrifuged, resuspended in the assay medium, and standardized to 10^9^ CFU/ml. Aliquots of 49 μl of this standardized suspension were added to the wells. Next, 1 μl solvent control or 1 μl of a 100-times concentrated solution of **6a** or **8b** was added to the test wells. Subsequently the plates were incubated at 25 °C for up to 2 days. The fraction of dead worms was determined by counting the number of dead worms and the total number of worms in each well, using an Evos FL auto Microscope (Life technologies). The compounds were tested at least six times in each assay and each assay was repeated at least two times (n ≥ 12). At least 25 *C. elegans* nematodes were used for in each well (n ≥ 300 nematodes/condition).

### Mammalian cell viability assay

Human cervical carcinoma-derived HeLa cells were maintained in routine culture with weekly splitting. Cells were cultured in DMEM medium supplemented with fetal bovine serum (10%), L-glutamine (1%), penicillin (100 U/ml), streptomycin (100 μg/ml) and maintained at 37 °C in 5% CO_2_ atmosphere. All reagents were purchased from Life Technologies. Twenty-four hours before drug treatment, cells were trypsinized, resuspended in medium and counted on a Z2 Beckman Coulter counter (Beckman Coulter) in order to obtain a confluent 12-wells plate. Different drug concentrations were tested as reported in each experiment and cell counting was performed respectively at 24 h and 48 h for each concentration. Cell viability data were derived from two independent experiments and were normalized to NT sample at 24 h. NT samples were subjected only to DMSO solvent. Microphotographs were obtained through an inverted microscope (Nikon) equipped with phase contrast and dark field illumination.

## Additional Information

**How to cite this article**: Scoffone, V. C. *et al*. Discovery of new diketopiperazines inhibiting *Burkholderia cenocepacia* quorum sensing *in vitro* and *in vivo*. *Sci. Rep.* **6**, 32487; doi: 10.1038/srep32487 (2016).

## Supplementary Material

Supplementary Information

## Figures and Tables

**Figure 1:**
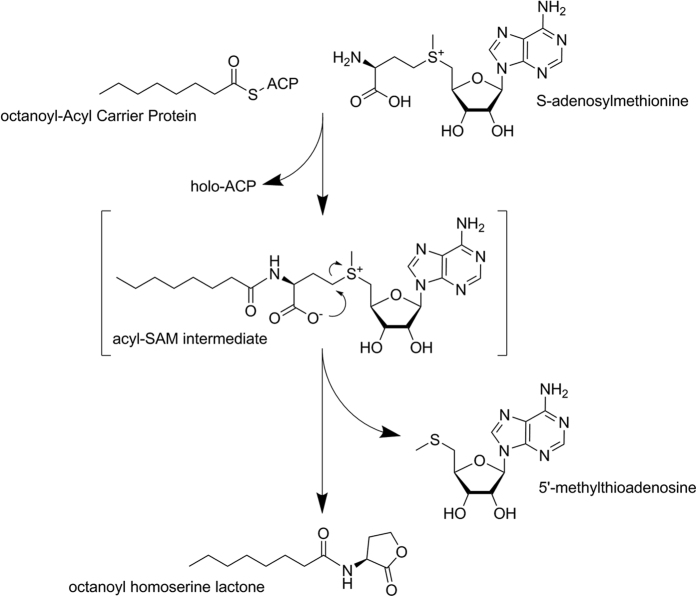
CepI catalyzed formation of homoserine lactone. The two substrates octanoyl-acyl carrier protein (ACP) and S-adenosylmethionine (SAM) form an acyl-SAM intermediate with the releasing of the holo-ACP. The subsequent lactonization gives rise to 5′-methylthioadenosine and the signal molecule octanoyl homoserine lactone.

**Table 1:** Percent survival of uninfected and infected *C. elegans* nematodes (average ± SD) receiving no treatment (CTRL) or a treatment with the compounds at different concentrations.

Compound	Concentration (μM)	*C. elegans* survival (%)			
		24 h		48 h	
		No infection	*B. cenocepacia* J2315	No infection	*B. cenocepacia* J2315
CTRL	—	96 ± 5	65 ± 7	95 ± 5	35 ± 18
**6a**	1	98 ± 6	73 ± 10*	91 ± 12	56 ± 11*
	5	99 ± 2	86 ± 6*	98 ± 4	66 ± 5*
	10	91 ± 8	88 ± 17*	86 ± 10	74 ± 11*
	25	91 ± 4	91 ± 9*	91 ± 6	82 ± 5*
**8b**	1	98 ± 5	84 ± 2*	97 ± 6	50 ± 6*
	5	97 ± 7	89 ± 4*	95 ± 7	55 ± 9*
	10	99 ± 2	93 ± 7*	95 ± 10	70 ± 7*
	25	98 ± 5	91 ± 5*	96 ± 5	75 ± 3*

The results are expressed as percentage survival after 24 h and 48 h of infection. *Survival of infected and treated *C. elegans* is significantly higher compared to the infected untreated controls (p < 0.05).

## References

[b1] Angelis A., Tordrup D. & Kanavos P. Socio-economic burden of rare diseases: a systematic review of cost of illness evidence. Health Policy. 119, 964–979 (2015). 25661982 10.1016/j.healthpol.2014.12.016

[b2] Döring G., Flume P., Heijerman H., Elborn J. S. & Group C. S. Treatment of lung infection in patients with cystic fibrosis: current and future strategies. J. Cyst. Fibros. 11, 461–479 (2012). 23137712 10.1016/j.jcf.2012.10.004

[b3] Ciofu O., Hansen C. R. & Høiby N. Respiratory bacterial infections in cystic fibrosis. Curr. Opin. Pulm. Med. 19, 251–258 (2013). 23449384 10.1097/MCP.0b013e32835f1afc

[b4] Frangolias D. D. . *Burkholderia cepacia* in cystic fibrosis. Variable disease course. Am. J. Respir. Crit. Care Med. 160, 1572–1577 (1999). 10556123 10.1164/ajrccm.160.5.9805046

[b5] Drevinek P. & Mahenthiralingam E. *Burkholderia cenocepacia* in cystic fibrosis: epidemiology and molecular mechanisms of virulence. Clin. Microbiol. Infect. 16, 821–830 (2010). 20880411 10.1111/j.1469-0691.2010.03237.x

[b6] Saiman L., Siegel J. & Foundation C. F. Infection control recommendations for patients with cystic fibrosis: microbiology, important pathogens, and infection control practices to prevent patient-to-patient transmission. Infect. Control. Hosp. Epidemiol. 24, S6–52 (2003). 12789902 10.1086/503485

[b7] Van Acker H. . Biofilm-grown *Burkholderia cepacia* complex cells survive antibiotic treatment by avoiding production of reactive oxygen species. PLoS One 8, e58943 (2013). 23516582 10.1371/journal.pone.0058943 PMC3596321

[b8] Tomlin K. L. . Quorum-sensing mutations affect attachment and stability of *Burkholderia cenocepacia* biofilms. Appl. Environ. Microbiol. 71, 5208–5218 (2005). 16151106 10.1128/AEM.71.9.5208-5218.2005 PMC1214635

[b9] Sokol P. A. . The CepIR quorum-sensing system contributes to the virulence of *Burkholderia cenocepacia* respiratory infections. Microbiology 149, 3649–3658 (2003). 14663096 10.1099/mic.0.26540-0

[b10] Sokol P. A., Malott R. J., Riedel K. & Eberl L. Communication systems in the genus *Burkholderia*: global regulators and targets for novel antipathogenic drugs. Future Microbiol. 2, 555–563 (2007). 17927476 10.2217/17460913.2.5.555

[b11] Rasko D. A. & Sperandio V. Anti-virulence strategies to combat bacteria-mediated disease. Nat. Rev. Drug Discov. 9, 117–128 (2010). 20081869 10.1038/nrd3013

[b12] Schuster M., Sexton D. J., Diggle S. P. & Greenberg E. P. Acyl-homoserine lactone quorum sensing: from evolution to application. Annu. Rev. Microbiol. 67, 43–63 (2013). 23682605 10.1146/annurev-micro-092412-155635

[b13] Suppiger A., Schmid N., Aguilar C., Pessi G. & Eberl L. Two quorum sensing systems control biofilm formation and virulence in members of the *Burkholderia cepacia* complex. Virulence 4, 400–409 (2013). 23799665 10.4161/viru.25338 PMC3714132

[b14] Boon C. . A novel DSF-like signal from *Burkholderia cenocepacia* interferes with *Candida albicans* morphological transition. ISME J. 2, 27–36 (2008). 18049456 10.1038/ismej.2007.76

[b15] Coenye T. Social interactions in the *Burkholderia cepacia* complex: biofilms and quorum sensing. Future Microbiol. 5, 1087–1099 (2010). 20632807 10.2217/fmb.10.68

[b16] Mahenthiralingam E., Urban T. A. & Goldberg J. B. The multifarious, multireplicon *Burkholderia cepacia* complex. Nat. Rev. Microbiol. 3, 144–156 (2005). 15643431 10.1038/nrmicro1085

[b17] Lewenza S., Conway B., Greenberg E. P. & Sokol P. A. Quorum sensing in *Burkholderia cepacia*: identification of the LuxRI homologs CepRI. J. Bacteriol. 181, 748–756 (1999). 9922236 10.1128/jb.181.3.748-756.1999 PMC93439

[b18] Udine C. . Phenotypic and genotypic characterisation of *Burkholderia cenocepacia* J2315 mutants affected in homoserine lactone and diffusible signal factor-based quorum sensing systems suggests interplay between both types of systems. PLoS One 8, e55112 (2013). 23383071 10.1371/journal.pone.0055112 PMC3557247

[b19] Christensen Q. H., Grove T. L., Booker S. J. & Greenberg E. P. A high-throughput screen for quorum-sensing inhibitors that target acyl-homoserine lactone synthases. Proc. Natl. Acad. Sci. USA 110, 13815–13820 (2013). 23924613 10.1073/pnas.1313098110 PMC3752275

[b20] Campbell J., Lin Q., Geske G. D. & Blackwell H. E. New and unexpected insights into the modulation of LuxR-type quorum sensing by cyclic dipeptides. ACS Chem. Biol. 4, 1051–1059 (2009). 19928886 10.1021/cb900165y PMC2801563

[b21] Raychaudhuri A., Jerga A. & Tipton P. A. Chemical mechanism and substrate specificity of RhlI, an acylhomoserine lactone synthase from *Pseudomonas aeruginosa*. Biochemistry 44, 2974–2981 (2005). 15723540 10.1021/bi048005m

[b22] Chung J. . Small-molecule inhibitor binding to an N-acyl-homoserine lactone synthase. Proc. Natl. Acad. Sci. USA. 108, 12089–12094 (2011). 21730159 10.1073/pnas.1103165108 PMC3141922

[b23] Watson W. T., Minogue T. D., Val D. L., von Bodman S. B. & Churchill M. E. Structural basis and specificity of acyl-homoserine lactone signal production in bacterial quorum sensing. Mol. Cell 9, 685–694 (2002). 11931774 10.1016/s1097-2765(02)00480-x

[b24] Gould T. A., Schweizer H. P. & Churchill M. E. Structure of the *Pseudomonas aeruginosa* acyl-homoserinelactone synthase LasI. Mol. Microbiol. 53, 1135–1146 (2004). 15306017 10.1111/j.1365-2958.2004.04211.x

[b25] Churchill M. E. & Chen L. Structural basis of acyl-homoserine lactone-dependent signaling. Chem. Rev. 111, 68–85 (2011). 21125993 10.1021/cr1000817 PMC3494288

[b26] Hanzelka B. L., Stevens A. M., Parsek, M. R., Crone T. J. & Greenberg, E. P. Mutational analysis of the *Vibrio fischeri* LuxI polypeptide: critical regions of an autoinducer synthase. J. Bacteriol. 179, 4882–4887 (1997). 9244278 10.1128/jb.179.15.4882-4887.1997 PMC179337

[b27] Parsek M. R., Schaefer A. L. & Greenberg E. P. Analysis of random and site-directed mutations in *rhII*, a *Pseudomonas aeruginosa* gene encoding an acylhomoserine lactone synthase. Mol. Microbiol. 26, 301–310 (1997). 9383155 10.1046/j.1365-2958.1997.5741935.x

[b28] Huber B. . The *cep* quorum-sensing system of *Burkholderia cepacia* H111 controls biofilm formation and swarming motility. Microbiology 147, 2517–2528 (2001). 11535791 10.1099/00221287-147-9-2517

[b29] Kooi C., Subsin B., Chen R., Pohorelic B. & Sokol P. A. *Burkholderia cenocepacia* ZmpB is a broad-specificity zinc metalloprotease involved in virulence. Infect. Immun. 74, 4083–4093 (2006). 16790782 10.1128/IAI.00297-06 PMC1489746

[b30] Schwyn B. & Neilands J. B. Universal chemical assay for the detection and determination of siderophores. Anal. Biochem. 160, 47–56 (1987). 2952030 10.1016/0003-2697(87)90612-9

[b31] Brackman G., Cos P., Maes L., Nelis H. J. & Coenye T. Quorum sensing inhibitors increase the susceptibility of bacterial biofilms to antibiotics *in vitro* and *in vivo*. Antimicrob. Agents Chemother. 55, 2655–2661 (2011). 21422204 10.1128/AAC.00045-11 PMC3101409

[b32] Clatworthy A. E., Pierson E. & Hung D. T. Targeting virulence: a new paradigm for antimicrobial therapy. Nat. Chem. Biol. 3, 541–548 (2007). 17710100 10.1038/nchembio.2007.24

[b33] Laxminarayan R. . Access to effective antimicrobials: a worldwide challenge. Lancet. 387, 168–175 (2016). 26603918 10.1016/S0140-6736(15)00474-2

[b34] Rajamanikandan S., Jeyaraman J. & Pappu S. Binding mode exploration of LuxR-thiazolidinedione analogues, e-pharmacophore based virtual screening in the designing of LuxR inhibitors and its biological evaluation. J. Biomol. Struct. Dyn. 4, 1–57 (2016). 27141809 10.1080/07391102.2016.1166455

[b35] Sethupathy S. . Proteomic analysis reveals modulation of iron homeostasis and oxidative stress response in *Pseudomonas aeruginosa* PAO1 by curcumin inhibiting quorum sensing regulated virulence factors and biofilm production. J. Proteomics S1874–3919, 30141–30145 (2016). 10.1016/j.jprot.2016.04.019 27108548

[b36] Tal-Gan Y., Ivancic M., Cornilescu G., Yang T. & Blackwell H. E. Highly stable, amide-bridged autoinducing peptide analogues that strongly inhibit the AgrC quorum sensing receptor in *Staphylococcus aureus*. Angew. Chem. Int. Ed. Engl (2016). 10.1002/anie.201602974 PMC4972187 27276693

[b37] Yang T., Tal-Gan Y., Paharik A. E., Horswill A. R. & Blackwell H. E. Structure-function analyses of a *Staphylococcus epidermidis* autoinducing peptide reveals motifs critical for AgrC-type receptor modulation. ACS Chem. Biol. (2016). 10.1021/acschembio.6b00120 PMC4946969 27159024

[b38] Darling P., Chan M., Cox A. D. & Sokol P. A. Siderophore production by cystic fibrosis isolates of *Burkholderia cepacia*. Infect. Immun. 66, 874–877 (1998). 9453660 10.1128/iai.66.2.874-877.1998 PMC107988

[b39] Lewenza S. & Sokol P. A. Regulation of ornibactin biosynthesis and N-acyl-homoserine lactone production by CepR in *Burkholderia cepacia*. J. Bacteriol. 183, 2212–2218 (2001). 11244059 10.1128/JB.183.7.2212-2218.2001 PMC95126

[b40] Sokol P. A., Darling P., Woods D. E., Mahenthiralingam E. & Kooi C. Role of ornibactin biosynthesis in the virulence of *Burkholderia cepacia*: characterization of *pvdA*, the gene encoding L-ornithine N(5)-oxygenase. Infect. Immun. 67, 4443–4455 (1999). 10456885 10.1128/iai.67.9.4443-4455.1999 PMC96763

[b41] Malott R. J. . A *Burkholderia cenocepacia* orphan LuxR homolog is involved in quorum-sensing regulation. J. Bacteriol. 191, 2447–2460 (2009). 19201791 10.1128/JB.01746-08 PMC2668411

[b42] Reuter K., Steinbach A. & Helms V. Interfering with bacterial quorum sensing. Perspect. Medicin. Chem. 8, 1–15 (2016). 26819549 10.4137/PMC.S13209 PMC4718088

[b43] Lowery C. A., Salzameda N. T., Sawada D., Kaufmann G. F. & Janda K. D. Medicinal chemistry as a conduit for the modulation of quorum sensing. J. Med. Chem. 53, 7467–7489 (2010). 20669927 10.1021/jm901742e PMC2974035

[b44] Lowery C. A. . Revisiting AI-2 quorum sensing inhibitors: direct comparison of alkyl-DPD analogues and a natural product fimbrolide. J. Am. Chem. Soc. 131, 15584–15585 (2009). 19824634 10.1021/ja9066783 PMC2784249

[b45] Zhu J. & Kaufmann G. F. Quo vadis quorum quenching? Curr. Opin. Pharmacol. 13, 688–698 (2013). 23876839 10.1016/j.coph.2013.07.003

[b46] Cronan J. E. & Thomas J. Bacterial fatty acid synthesis and its relationships with polyketide synthetic pathways. Methods Enzymol. 459, 395–433 (2009). 19362649 10.1016/S0076-6879(09)04617-5 PMC4095770

[b47] Quadri L. E. . Characterization of Sfp, a *Bacillus subtilis* phosphopantetheinyl transferase for peptidyl carrier protein domains in peptide synthetases. Biochemistry 37, 1585–1595 (1998). 9484229 10.1021/bi9719861

[b48] Rock C. O. & Cronan J. E. Acyl-acyl carrier protein synthetase from *Escherichia coli*. Methods Enzymol. 71 Pt C, 163–168 (1981). 7024728 10.1016/0076-6879(81)71023-1

[b49] Copeland A. Enzymes: A Practical Introduction to Structure, Mechanism, and Data Analysis. 2nd edn, (John Wiley & Sons Inc., 2000).

[b50] Brackman G. . Thiazolidinedione derivatives as novel agents against *Propionibacterium acnes* biofilms. J. Appl. Microbiol. 116, 492–501 (2014). 24251377 10.1111/jam.12378

[b51] Cooper V. S., Carlson W. A. & Lipuma J. J. Susceptibility of *Caenorhabditis elegan*s to *Burkholderia* infection depends on prior diet and secreted bacterial attractants. PLoS One 4, e7961 (2009). 19956737 10.1371/journal.pone.0007961 PMC2776534

[b52] Stiernagle T. Maintenance of C. elegans in WormBook 1–11 (2006). 18050451 10.1895/wormbook.1.101.1 PMC4781397

