# Supplementary material for: Discovery of new diketopiperazines inhibiting Burkholderia cenocepacia quorum sensing in vitro and in vivo
Source: Sci Rep. 2016 Sep 1;6:32487. doi: 10.1038/srep32487 (PMC5007513; doi:10.1038/srep32487)
Supplement: Supplementary Information [file srep32487-s1.pdf]

Supplementary Information

Discovery of new diketopiperazines inhibiting *Burkholderia cenocepacia* quorum sensing *in vitro* and *in vivo*

Viola C. Scoffone[1,+], Laurent R. Chiarelli[1,+], Vadim Makarov[2,+,*], Gilles Brackman[3], Aygun Israyilova[1,4], Alberto Azzalin[5,6], Federico Forneris[1], Olga Riabova[2], Svetlana Savina[2], Tom Coenye[3], Giovanna Riccardi[1] and Silvia Buroni[1,*].

[1]Dipartimento di Biologia e Biotecnologie, Università degli Studi di Pavia, Via Ferrata, 1-27100 Pavia, Italy;
[2]Lab for Biomedicinal Chemistry, Bach Institute of Biochemistry, Research Center of Biotechnology of the Russian Academy of Sciences, Moscow 119071, Russia;
[3]Lab of Pharmaceutical Microbiology, Ghent University, Ottergemsesteenweg 460, Ghent, 9000, Belgium;
[4]Department of Microbiology, Baku State University, Z. Khalilov 23, AZ1148, Baku, Azerbaijan;
[5]Neurosurgery, Dipartimento di Scienze Clinico-Chirurgiche e Pediatriche, Università degli Studi di Pavia, Fondazione IRCCS Policlinico S. Matteo, Pavia, Italy;
[6] IGM-CNR, Via Abbiategrasso 207, 27100, Pavia, Italy.

*Correspondence and requests for materials should be addressed V.M. (email: makar-cl@ropnet.ru) and to S.B. (email: silvia.buroni@unipv.it).
[+]these authors contributed equally to this work

Supplementary information contents:

- Supplementary Figures S1 to S6
- Supplementary Table S1 and S2
- Supplementary Methods
- Supplementary References

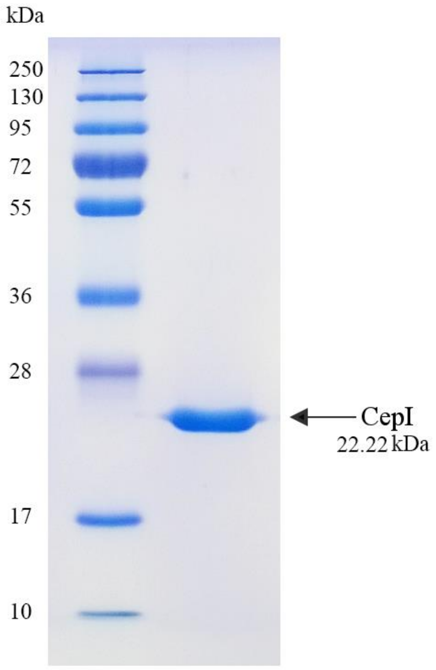

Supplementary Figure S1. SDS-PAGE of CepI. SDS-PAGE of CepI after purification on HiLoad 26/60 Superdex 200 size exclusion column (GE Healthcare). The arrow indicates the CepI band and its molecular weight in kDa.

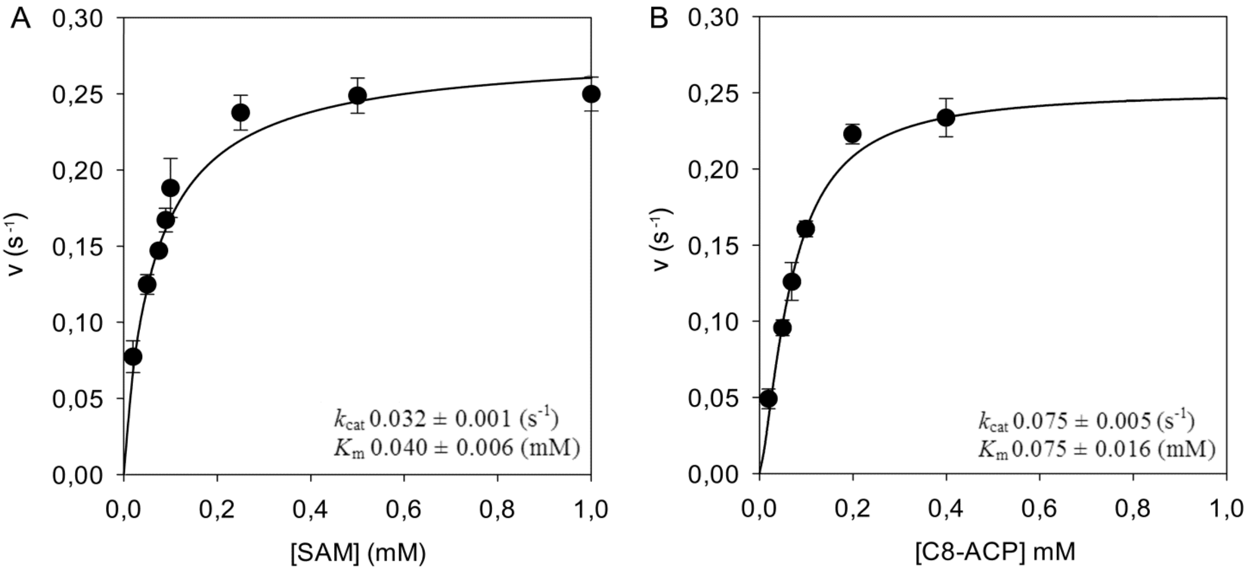

Supplementary Figure S2. Steady state kinetics of CepI. Steady state kinetics of CepI as a function of S-adenosyl methionine at fixed 0.1 mM C8-ACP concentration (A) and as a function of C8-ACP at fixed 1 mM S-adenosyl methionine (B). All experiments were performed at 37 °C as reported in the ‘‘Material and Methods’’ section.

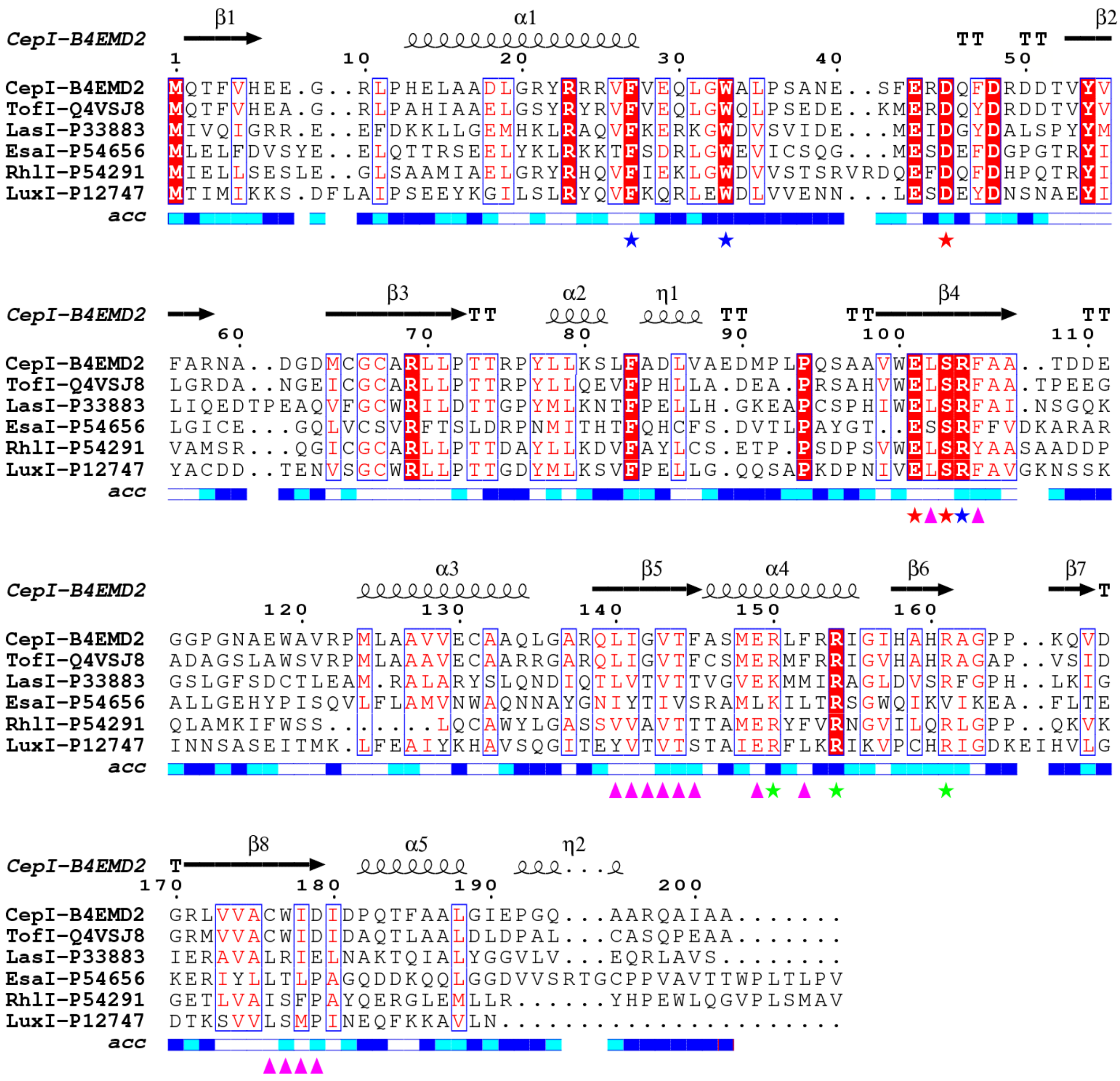

Supplementary Figure S3. Sequence alignment of CepI with related homologous acyl homoserine lactone synthase. Sequences have been selected based on availability of biochemical characterization and/or three-dimensional structures. Uniprot unique sequence identifiers are shown next to protein names. The alignment (generated with MUSCLE [S1]) highlights the conservation of predicted secondary structure features, the amino acid sequence and their solvent accessibility (*acc*, shown in a scale from white to blue to indicate highly and poorly solvent accessible, respectively) based on the CepI homology model. Critical residues for enzymatic activity are shown with a star symbol, either in red (highly conserved residues essential for catalysis), in blue (residues implicated in stabilization of the SAM substrate), or in green (positively charged residues implicated in binding and stabilization of the ACP substrate). Residues lining the putative hydrophobic cavity hosting the acyl moiety of AHL are shown with pink triangles.

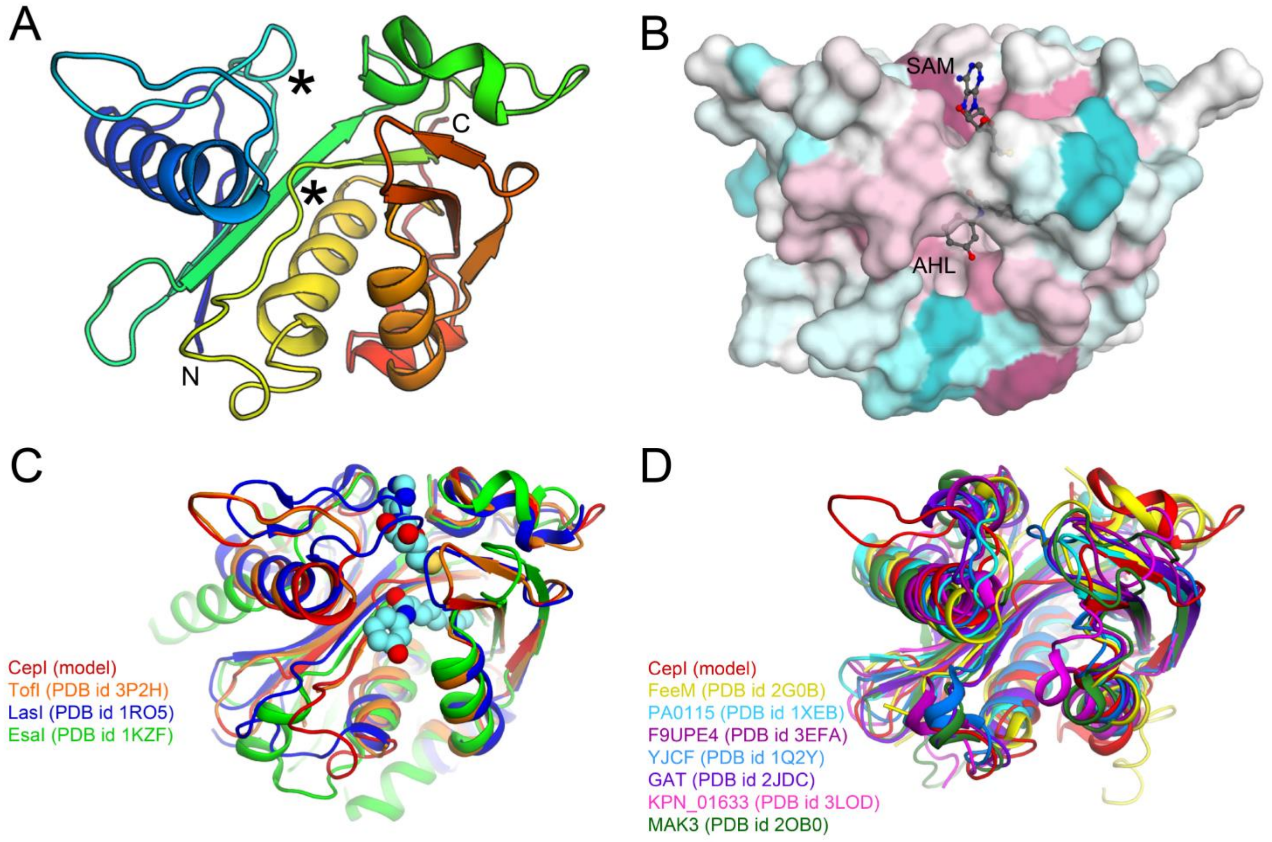

Supplementary Figure S4. **Homology modelling of CepI and comparison with related structural homologs.** (A) Three-dimensional surface and cartoon representation of the CepI homology model. The structure is colored from the N-terminus (blue) to the C-terminus (red) of the molecule. The substrate binding sites identified by comparison with structural homologs are highlighted with asterisks. (B) CONSURF[S2] representation of amino acid sequence conservation of CepI with related AHL synthases, based on the sequence alignment shown in Supplementary Fig. S1. For better identification of the common substrate binding cavities, the substrate intermediate MTA and the acyl-like J8-C8 inhibitor identified in the TofI structure[S3] (PDB id 3P2H) are shown in the superposition as grey ball-and sticks. (C) Superposition of CepI homology model with acetylhomoserine lactone synthases of known three-dimensional structure. The MTA intermediate and the J8-C8 inhibitor identified in the TofI structure[S3] are superimposed as in panel B, and shown as cyan spheres. (D) Superposition of CepI homology model with other N-acyltransferases sharing sequence and structure homology identified by HHPRED analysis[S4].

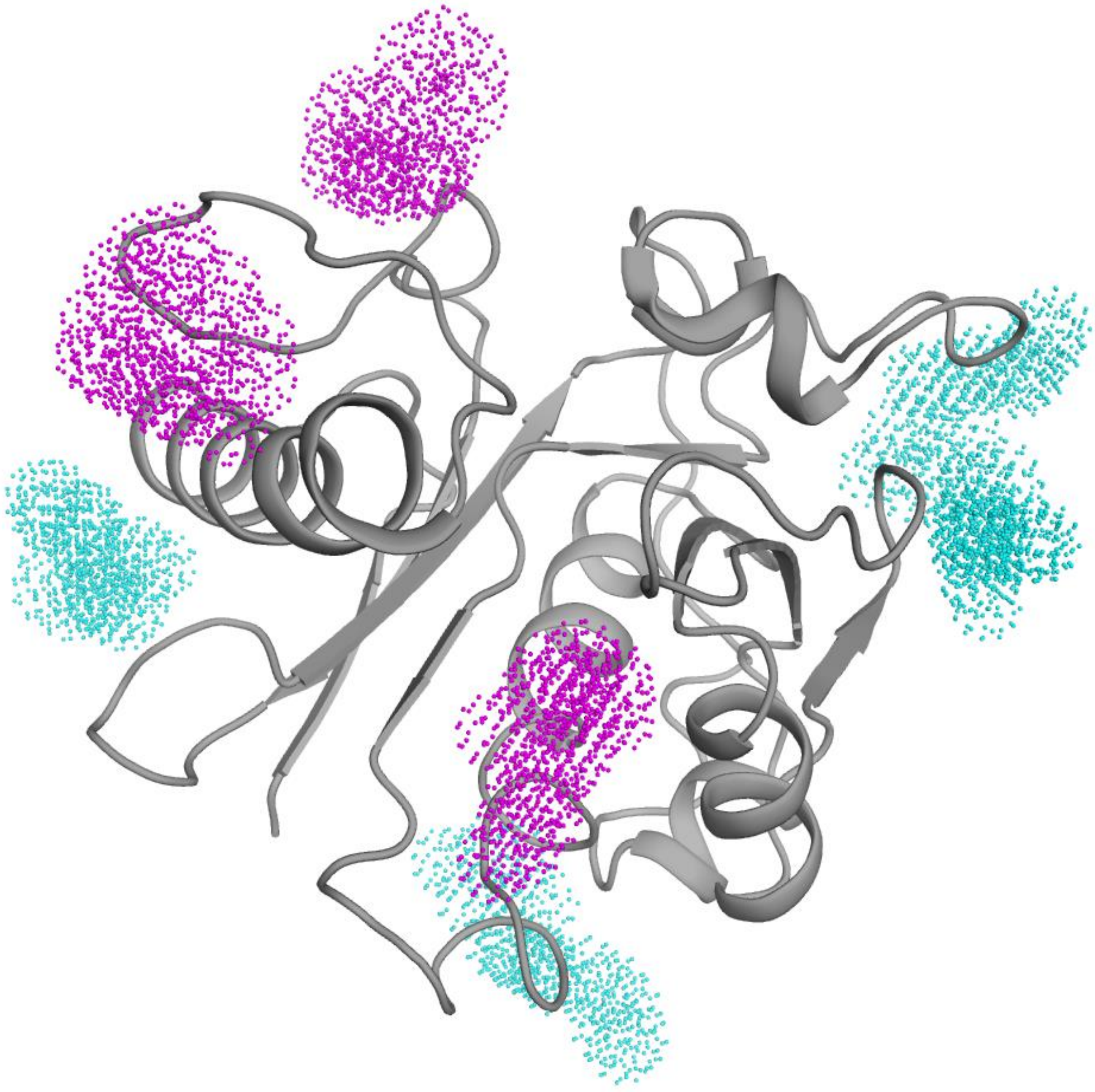

Supplementary Figure S5. **Molecular docking analysis of compound 8b onto CepI structure.** Cartoon representation of the CepI homology model (shown in grey, orientation as in Supplementary Figure S4) showing the results of molecular docking using compound **8b**. Optimized candidate ligand binding sites identified by molecular docking are shown with colored dots. Candidate sites proximate to structural elements critical for substrate recognition are shown in magenta, others in cyan.

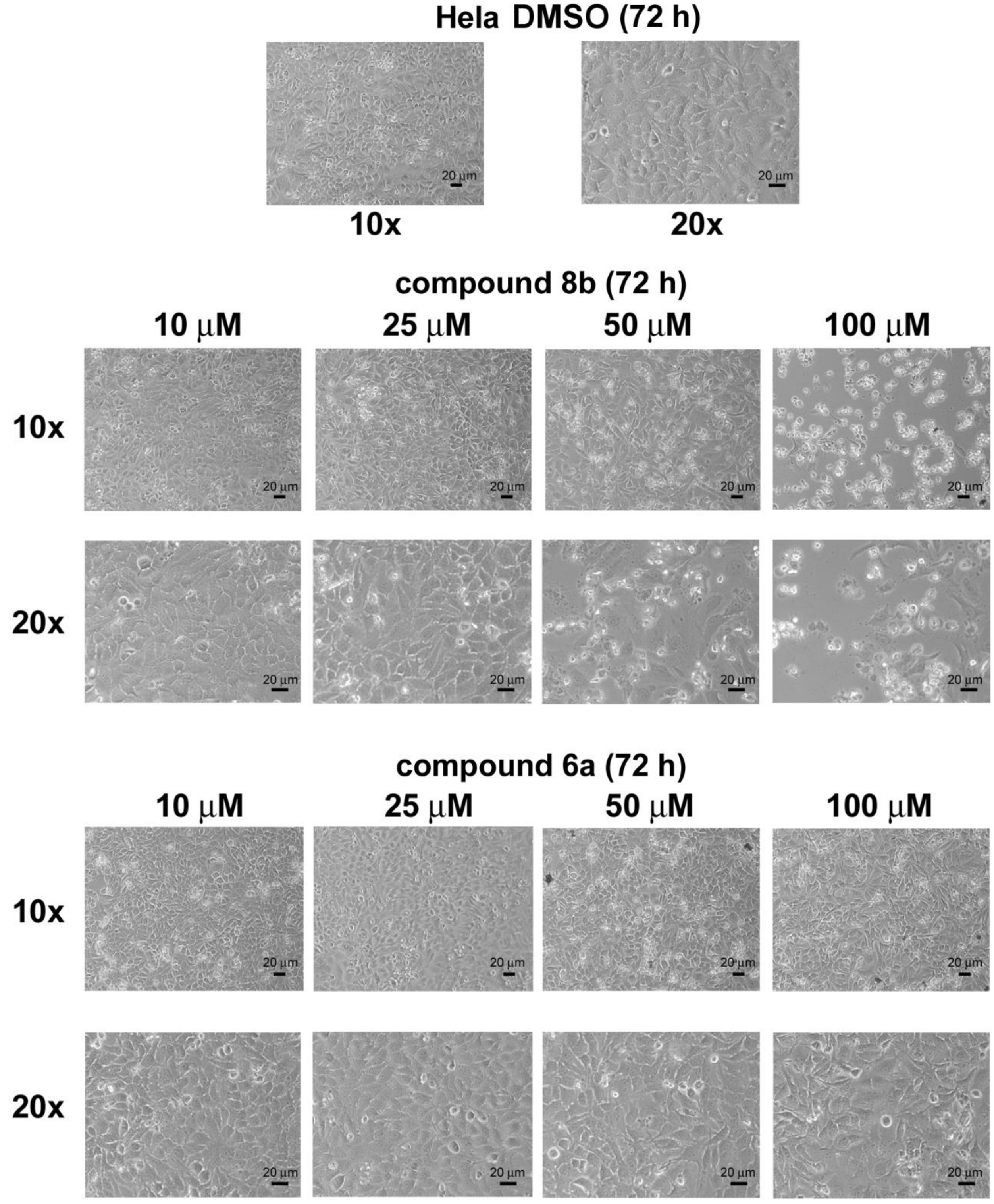

Supplementary Figure S6. Microphotographs of HeLa cells after 72h treatment with 8b and 6a. DMSO, cells treated with the highest amount of solvent. 10X and 20X indicate the magnification used.

Supplementary Table S1. Biological and biochemical characterization of diketopiperazines.

Compound	Structure	CepI IC_{50} (mM)	MIC against *B. cenocepacia* (μg/ml)
6a<br>10826023	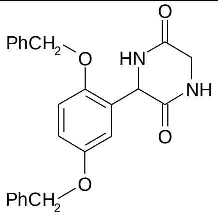	0.030±0.006	> 128
6b<br>10826027	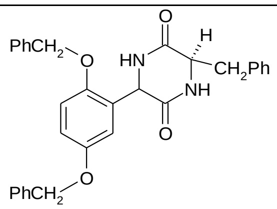	not inhibitory	> 128
6c<br>10826028	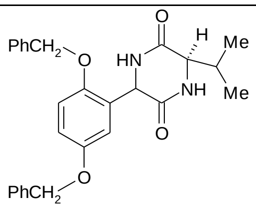	not inhibitory	> 128
7a<br>10826029	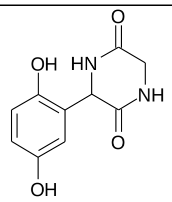	not inhibitory	>128
7b<br>10826031	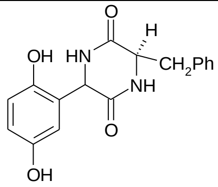	not inhibitory	> 128
7c<br>10826032	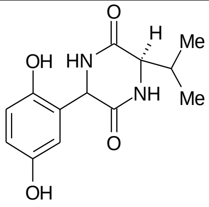	not inhibitory	> 128
8a<br>11626106	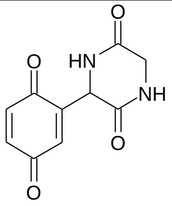	0.010±0.001	>128
8b<br>11526011	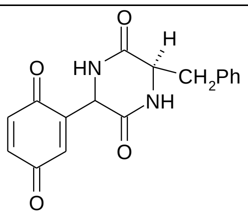	0.005±0.0008	> 128

8c<br>11626107	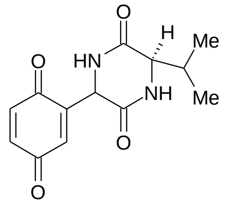	0.006±0.0006	>128
9<br>11526012	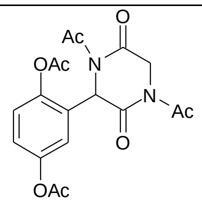	not inhibitory	> 128

Supplementary Table S2: Structural folds related to CepI identified by HHPRED analysis[S4].

Protein	PDB id	seq. id.	Reference
TofI from *B. glumae*	3P2H	67%	Chung, J. *et al*., (2011)[S3].
LasI from *P. aeruginosa*	1RO5	28%	Gould, T. A. *et al*., (2004)[S5].
EsaI from *E. stewardii*	1KZF	18%	Watson W. T. *et al*., (2002)[S6].
FeeM from uncultured soil microbe	2G0B	15%	Van Wagoner R. M. *et al*., (2006)[S7].
PA0115 from *P. aeruginosa*	1XEB	17%	N/A
F9UPE4 from *L. plantarum*	3EFA	23%	N/A
YJCF from *B. subtilis*	1Q2Y	17%	N/A
GAT from *B. licheniformis*	2JDC	13%	Siehl, D. L. *et al*., (2007)[S8].
KPN_01633 from *K. pneumoniae*	3LOD	17%	N/A
MAK3 from *H. Sapiens*	2OB0	10%	N/A

Supplementary Methods

Structural bioinformatics

Secondary structure prediction on CepI was performed through comparison of the results of multiple web servers including HHPRED[S4] and PredictProtein[S9]. The comparative evaluation of multiple structural prediction algorithms enabled unambiguous identification of the most prominent features of the proteins analyzed. The three-dimensional model of CepI was created using the homology modelling tools in HHPRED[S4] and MODELLER[S10], and the alignment shown in Supplementary Fig. S1. The model was further optimized by geometry idealization using PHENIX[S11]. Final model quality was assessed using PROCHECK and PDBSUM[S12] and the Qmean server[S13]. Structural figures were generated using ESPript[S14] and PyMol[S15].

Molecular Docking

Molecular docking of compound **8b** was performed using the CepI homology model obtained as described above and the three-dimensional structure of the inhibitor generated by the PRODRG server[S16]. Unconstrained ligand exploration trials for identification of candidate ligand binding sites were performed by repeated searches using the SWISSDOCK[S17] and PELE[S18] docking servers. Out of more than 100 parallel independent attempts of unconstrained ligand binding search, the best candidate sites were selected based on the lowest stabilization energy and used as templates for ligand binding refinement using PELE[S19]. Each candidate binding contact was subject to 24 parallel runs of refinement (using 12 hours of minimization for each run). Final selection used to generate Supplementary Figure S4 included ligand-bound conformations showing the lowest stabilization energies and ligand mobility.

General procedure for the synthesis diketopiperazine derivatives (Figure 8 in main text)

Step a). Suspension of 2',5'-Dihydroxyacetophenone (3.0 g, 19,7 mmol), benzyl chloride (4.5 ml, 39.4 mmol) and potassium carbonate (8.2 g, 59.1 mmol) in 75 ml of abs. ethanol was refluxed 4 hours, filtered off and mother liquid was evaporated under vacuum, residue treated by cold water and white solid was filtered off. Crude 2',5'-dibenzyloxyacetophenone was recrystallized from ethanol. The yield is 5.05 g (77%), mp. 77-78 ^{o}C (lit. 76-77 ^{o}C)[S20].

Step b). Suspension of 2',5'-dibenzyloxyacetophenone (3.0 g, 9.0 mmol) and selenium dioxide (1.63 g, 14.85 mmol) in pyridine (25 ml) was heated for 100 ^{o}C for 3 hours, filtered off and mother liquid was evaporated under vacuum, residue was solved in 5% NaOH water solution. This solution was washed by ester (3 x 30 ml) from unreacted starting material and treated by 5% hydrochloride

acid. Formed white precipitate was filtered off, washed by water and recrystallized from mixture ethanol/water. The yield of [2,5-bis(benzyloxy)phenyl](oxo)acetic acid is 2.64 g (81%).

[2,5-Bis(benzyloxy)phenyl](oxo)acetic acid **3**. Mp. 163-5 ºC (EtOH). LCMS (ESI): *m/z* 363.3754 $(M+H)^+$. $C_{22}H_{18}O_5$. ^1H NMR (DMSO-d_6): 5.07 (s, 4H, 2 OCH_2), 7.05-7.45 (m, 13H, 3CH+2Ph), ppm.

Step c). Suspension of [2,5-bis(benzyloxy)phenyl](oxo)acetic acid (2.5 g, 6.9 mmol) in 15 ml of carbon tetrachloride was treated by 2.5 ml of thionyl chloride and one drop of DMF. Reaction mixture was refluxed for 3 hours become clear solution and evaporated under vacuum. Oily residue was treated by toluene and evaporated under vacuum again. Colorless oil of [2,5-bis(benzyloxy)phenyl](oxo)acetyl chloride (quantitative yield) was immediately used in next step without any additional purification.

Step d). Solution of [2,5-bis(benzyloxy)phenyl](oxo)acetyl chloride (6.9 mmol) from previous step in 10 ml of dry DMF was treated by corresponding amino ester hydrochloride (7.0 mmol) and triethylamine (1.9 ml, 13.8 mmol). Reaction mixture was stored for 48 hours at room temperature on magnetic stirrer and evaporated under vacuum. Residue was treated by 10 ml of water, formed white precipitate was filtered off, washed by water and recrystallized from mixture ethanol/water.

Methyl [[[2,5-bis(benzyloxy)phenyl](oxo)acetyl]amino]acetate **5a**. Yield 51%. Mp. 113-6 ºC (EtOH/water). LCMS (ESI): *m/z* 434.4533 $(M+H)^+$. $C_{26}H_{23}NO_6$. ^1H NMR (DMSO-d_6): 3.63 (s, 3H, OCH_3), 3.71 (d, 2H, J = 3.6 Hz, $NH\underline{CH_2}$), 5.08 (s, 2H, $O\underline{CH_2}$), 5.11 (s, 2H, $O\underline{CH_2}$), 7.10-7.40 (m, 13H, 3CH+2Ph), 8.96 (br s, 1H, NH) ppm.

Methyl N-[[2,5-bis(benzyloxy)phenyl](oxo)acetyl]-D-phenylalaninate **5b**. Yield 64%. Mp. 98-100 ºC (EtOH/water). LCMS (ESI): *m/z* 524.5758 $(M+H)^+$. $C_{32}H_{29}NO_6$. ^1H NMR (DMSO-d_6): 3.11 (d, 2H, J = 7.3 Hz, CH_2), 3.63 (s, 3H, OCH_3), 3.73 (t, 1H, J = 5.8 Hz, $NH\underline{CH}$), 5.09 (s, 2H, $O\underline{CH_2}$), 5.13 (s, 2H, $O\underline{CH_2}$), 7.10-7.40 (m, 18H, 3CH+3Ph), 8.98 (br s, 1H, NH) ppm.

Methyl N-[[2,5-bis(benzyloxy)phenyl](oxo)acetyl]-D-valinate **5c**. Yield 76%. Mp. 127-9 ºC (EtOH/water). LCMS (ESI): *m/z* 476.5330 $(M+H)^+$. $C_{28}H_{29}NO_6$. ^1H NMR (DMSO-d_6): 0.94 (dd, 6H, J = 7.0 Hz, 2 CH_3), 2.15 (m, 1H, $CH_3\underline{CH}CH_3$), 3.66 (s, 3H, OCH_3), 4.82 (d, 1H, J = 4.6 Hz, $NH\underline{CH_2}$), 5.10 (s, 2H, $O\underline{CH_2}$), 5.11 (s, 2H, $O\underline{CH_2}$), 7.10-7.40 (m, 13H, 3CH+2Ph), 8.91 (br s, 1H, NH) ppm.

Step e). Solution of corresponding methyl [[[2,5-bis(benzyloxy)phenyl](oxo)acetyl]-amino]acetate (3 mmol) in 25 ml of 10% solution of ammonia in ethanol was heated in bomb for 48 hours at 150 °C. After cooling solution was evaporated and residue of 3-[2,5-bis(benzyloxy)phenyl]-6-R-piperazine-2,5-dione recrystallized from ethanol.

3-[2,5-Bis(benzyloxy)phenyl]piperazine-2,5-dione **6a**. Yield 59%. Mp. 226-7 ºC. LCMS (ESI): *m/z* 403.4426 $(M+H)^+$. $C_{24}H_{22}N_2O_4$. ^1H NMR (DMSO-d_6): 3.61 (s, 2H, CH_2), 4.83 (s, 1H, CH), 5.09 (s, 2H, O$\underline{CH_2}$), 5.13 (s, 2H, O$\underline{CH_2}$), 7.05-7.40 (m, 13H, 3CH+2Ph), 8.01 (br s, 1H, NH), 8.12 (br s, 1H, NH) ppm.

(3S)-3-Benzyl-6-[2,5-bis(benzyloxy)phenyl]piperazine-2,5-dione **6b**. Yield 36%. Mp. 214-7 ºC. LCMS (ESI): *m/z* 494.5651 $(M+H)^+$. $C_{31}H_{28}N_2O_4$. ^1H NMR (DMSO-d_6): 2.65 (d, 2H, J = 69 Hz, CH_2), 3.97 (s, 1H, CH), 5.03 (s, 1H, CH), 5.10 (s, 2H, O$\underline{CH_2}$), 5.13 (s, 2H, O$\underline{CH_2}$), 7.05-7.45 (m, 18H, 3CH+3Ph), 8.14 (br s, 2H, 2NH) ppm.

(6S)-3-[2,5-Bis(benzyloxy)phenyl]-6-isopropylpiperazine-2,5-dione **6c**. Yield 68%. Mp. 198-200 ºC. LCMS (ESI): *m/z* 445.5223 $(M+H)^+$. $C_{27}H_{28}N_2O_4$. ^1H NMR (DMSO-d_6): 0.85 (dd, 6H, J = 7.0 Hz, 2 CH_3), 2.10 (m, 1H, $CH_3\underline{CH}CH_3$), 3.77 (s, 1H, CH), 5.06 (s, 2H, O$\underline{CH_2}$), 5.09 (s, 2H, O$\underline{CH_2}$), 5.13 (s, 1H, CH), 7.15-7.40 (m, 13H, 3CH+2Ph), 8.00 (br s, 1H, NH), 8.16 (br s, 1H, NH) ppm.

Step f). Solution of 3-[2,5-bis(benzyloxy)phenyl]-6-R-piperazine-2,5-dione (2 mmol) in 15 mlof DMF was treated by H_2 in presence of 0.1 g of 10% palladium on carbon for 4 hours at room temperature. Solution was filtered off, mother liquid was evaporated under vacuum and residue was treated by small volume of cold water. Formed white precipitate of 3-(2,5-dihydroxyphenyl)-6-R-piperazine-2,5-dione was filtered off, washed by cold water and recrystallized from water.

3-(2,5-Dihydroxyphenyl)piperazine-2,5-dione **7a**. Yield 59%. Mp. 194-7 ºC. LCMS (ESI): *m/z* 223.1975 $(M+H)^+$. $C_{10}H_{10}N_2O_4$. ^1H NMR (DMSO-d_6): 3.61 (q, 2H, J = 19.2 Hz, CH_2), 4.83 (s, 1H, CH), 6.57 (m, 3H, 3CH), 8.06 (br s, 1H, NH), 8.14 (s, 1H, NH), 8.43 (br s, 1H, OH), 9.01 (s, 1H, O), ppm.

(3S)-3-Benzyl-6-(2,5-dihydroxyphenyl)piperazine-2,5-dione **7b**. Yield 76%. Mp. 168-71 ºC. LCMS (ESI): *m/z* 313.3200 $(M+H)^+$. $C_{17}H_{16}N_2O_4$. ^1H NMR (DMSO-d_6): 3.04 (d, 2H, J = 71.2 Hz, CH_2), 4.33 (m, 1H, CH), 4.94 (s, 1H, CH), 6.61 (m, 3H, 3CH), 7.46 (m, 5H, Ph), 8.05 (br s, 1H, NH), 8.11 (s, 1H, NH), 8.23 (br s, 1H, OH), 8.87 (s, 1H, OH) ppm.

(6S)-3-(2,5-Dihydroxyphenyl)-6-isopropylpiperazine-2,5-dione **7c**. Yield 68%. Mp. 186-7 ºC (EtOH). LCMS (ESI): *m/z* 264.2772 $(M+H)^+$. $C_{13}H_{16}N_2O_4$. ^1H NMR (DMSO-d_6): 0.91 (dd, 6H, J = 7.0 Hz, 2 CH_3), 2.23 (m, 1H, $CH_3\underline{CH}CH_3$), 3.74 (s, 1H, CH), 4.95 (s, 1H, CH), 6.63 (m, 3H, 3CH), 8.00 (br s, 1H, NH), 8.09 (s, 1H, NH), 8.41 (br s, 1H, OH), 8.85 (s, 1H, OH) ppm.

Step g). Suspension of 3-(2,5-dihydroxyphenyl)-6-R-piperazine-2,5-dione (2.0 mmol) in 1.5 ml acetic acid was treated by 0.1 ml of perchloric acid 70% solution in water, 0.2 ml of conc. sulfuric acid and 0.15 ml of fum. nitric acid. After 15 minutes reaction mixture become yellow solution and after 3 hours yellow precipitate formatted. Reaction mixture was dissolved by 3 ml of isopropyl alcohol, stored at -18 ^oC for 1 hour, filtered off and washed by 2 ml of cold isopropyl alcohol. Solid

3-(3,6-dioxocyclohexa-1,4-dien-1-yl)-6-R-piperazine-2,5-dione was immediately recrystallized from ethanol.

3-(3,6-dioxocyclohexa-1,4-dien-1-yl)piperazine-2,5-dione **8a**. Yield 43%. Mp. 203-6 ºC. LCMS (ESI): *m/z* 221.1816 $(M+H)^+$. $C_{10}H_8N_2O_4$. ^1H NMR (DMSO-d_6): 3.73 (s, 2H, CH_2), 4.87 (s, 1H, CH), 6.65 (s, 1H, CH), 7.78 (s, 2H, CHCH), 8.21 (br s, 2H, 2 NH) ppm.

(3S)-3-Benzyl-6-(3,6-dioxocyclohexa-1,4-dien-1-yl)piperazine-2,5-dione **8b**. Yield 41%. Mp. 246-8 ºC. LCMS (ESI): *m/z* 311.3041 $(M+H)^+$. $C_{17}H_{14}N_2O_4$. ^1H NMR (DMSO-d_6): 2.27 (d, 2H, J = 69 Hz, CH_2), 3.98 (s, 1H, CH), 5.02 (s, 1H, CH), 6.73 (s, 2H, CHCH), 7.06 (m, 4H, Ph + CH), 7.53 (m, 2H, Ph), 7.94 (br s, 1H, NH), 8.43 (br s, 1H, NH) ppm.

(6S)-3-(3,6-Dioxocyclohexa-1,4-dien-1-yl)-6-isopropylpiperazine-2,5-dione **8c**. Yield 44%. Mp. 230-2 ºC (EtOH). LCMS (ESI): *m/z* 263.2613 $(M+H)^+$. $C_{13}H_{14}N_2O_4$. ^1H NMR (DMSO-d_6): 0.91 (dd, 6H, J = 7.0 Hz, 2 CH_3), 2.17 (m, 1H, $CH_3\underline{CH}CH_3$), 3.71 (s, 1H, CH), 4.83 (s, 1H, CH), 6.87 (s, 2H, 3CH), 8.16 (br s, 1H, NH), 8.28 (br s, 1H, NH) ppm.

Step h). Suspension of 3-(2,5-dihydroxyphenyl)piperazine-2,5-dione (0.7 g, 3.1 mmol) in 5 ml of acetic anhydride was refluxed for 6 hours. Reaction masse was evaporated and oily residue was recrystallized from ethanol. The yield of 2-(1,4-diacetyl-3,6-dioxopiperazin-2-yl)-1,4-phenylene diacetate 1.10 g (89%).

2-(1,4-Diacetyl-3,6-dioxopiperazin-2-yl)-1,4-phenylene diacetate **9**. Yield 89%. Mp. 149-51 ºC. LCMS (ESI): *m/z* 391.3442 $(M+H)^+$. $C_{18}H_{18}N_2O_8$. ^1H NMR (DMSO-d_6): 2.12, 2,16, 2.34, 2.48 (4s, 12 H, 4 $COCH_3$), 4.51 (d, 2H, J = 15.8 Hz, CH_2), 6.26 (s, 1H, CH), 7.18 (m, 3H, 3 CH) ppm.

Preparation of recombinant Sfp and ACP proteins

ACP and Sfp were amplified from *E. coli* BL21(DE3) and *B. subtilis* PB1927 genomic DNA respectively, using ACPfor (5’-ATGAGCACTATCGAAGAACGC-3’) and ACPrev (5’-TTACGCCTGGTGGCCGTTGAT-3’) and Sfpfor (5’-ATGAAGATTTACGGAATTTAT-3’) and Sfprev (5’-TTATAAAAGCTCTTCGTACGA-3’) primers. Fragments obtained were cloned into pETSUMO (Invitrogen).

ACP expression was obtained in *E. coli* BL21(DE3) cells by 3 h of induction with 0.5 mM IPTG at 37 °C. After induction, cells were resuspended in 100 mM TrisHCl pH 8.0, 1 mM DTT, 1:100 Protease Inhibitor Cocktail (Sigma-Aldrich), 1:100 5 mg/ml DNase (PanReac AppliChem) and disrupted by sonication. The cell-free extract obtained as above was incubated with 25 mM $MgCl_2$ and 1.2 mM $MnSO_4$ for 4 h at 37 °C to convert all ACP to the apo form[S21]. NaCl was added at a final concentration of 300 mM, and the cell-free extract was applied onto a HisTrap column, washed with 20 mM imidazole and the protein eluted with 250 mM imidazole. Tag cleavage was

achieved as described above. The protein was then dialyzed against 100 mM TrisHCl pH 8.0, 0.5 mM DTT and further purified by anionexchange chromatography on a Q-sepharose column (20 ml, GE-Healthcare), equilibrated in 100 mM TrisHCl pH 8.0, 0.5 mM DTT. Elution was performed using a 60 ml 0-1 M LiCl linear gradient. The purified protein was finally dialyzed against 100 mM TrisHCl pH 8.0, 0.5 mM DTT, concentrated to 17 mg/ml and stored at -80 °C until use. Protein concentration was evaluated by absorbance at 280 nm (ε=1490 M^{-1} cm^{-1}).

Recombinant Sfp was expressed in *E. coli* BL21(DE3) by 4 h induction with 0.5 mM IPTG at 37 °C. After induction, cells were resuspended in 50 mM TrisHCl pH 8.0, 300 mM NaCl, 5% glycerol, 5 mM imidazole, 1 mM DTT, 1:100 Protease Inhibitor Cocktail (Sigma-Aldrich), 1:100 5 mg/ml DNase (PanReac AppliChem), and disrupted by sonication. The cell-free extract, obtained as above, was applied onto an HisTrap column, washed with 20 mM imidazole and the protein eluted with 250 mM imidazole. Tag cleavage was obtained as described above. The protein was concentrated and loaded onto a HiLoad 26/60 Superdex 200 size exclusion column (GE Healthcare), equilibrated in 50 mM TrisHCl pH 8.0, 150 mM NaCl, 5% glycerol, 1 mM DTT. The purified protein was finally concentrated to 3 mg/ml and stored at -80 °C until use. Protein concentration was evaluated by absorbance at 280 nm (ε=28880 M^{-1} cm^{-1}).

MIC determination

The MICs (Minimal Inhibitory Concentrations) of the compounds reported in Supplementary Table S1 were assessed against *B. cenocepacia* J2315 by using the 2-fold microdilution method in U-bottom 96-well microtiter plates.

About 10^5 CFU were used to inoculate each well of the microplate containing concentrations of compounds ranging from 1 to 128 µg/ml. Growth was determined by the resazurin method[S22] after two days of incubation at 37 °C. 30 µl of a solution of resazurin sodium salt (Sigma Aldrich) at 0.01% in distilled water were added to each well, and the microtiters were incubated at 37 °C for about 4 h. The MIC was defined as the lowest concentration of the compound that prevented a change in color from blue to pink.

Synergistic experiments were performed using 20 µg/ml of **6a** or **8b** in combination with two-fold dilutions of ampicillin (1000-7.8 µg/ml), aztreonam (1000-7.8 µg/ml), ceftazidime (1000-7.8 µg/ml), chloramphenicol (32-0.25 µg/ml), ciprofloxacin (32-0.25 µg/ml), gentamicin (1000-7.8 µg/ml), kanamycin (1000-7.8 µg/ml), levofloxacin (32-0.25 µg/ml), meropenem (256-2 µg/ml), nalidixic acid (64-0.5 µg/ml), norfloxacin (256-2 µg/ml), sparfloxacin (64-0.5 µg/ml), streptomycin (1000-7.8 µg/ml), tetracycline (512-4 µg/ml), tobramycin (1000-7.8 µg/ml), trimethoprim (1000-7.8 µg/ml). The experiments were performed by the 2-fold microdilution method as described above.

Supplementary References

S1. Edgar, R. C. MUSCLE: multiple sequence alignment with high accuracy and high throughput. *Nucleic Acids Res.* **32**, 1792-1797 (2004).

S2. Ashkenazy, H., Erez, E., Martz, E., Pupko, T. & Ben-Tal, N. ConSurf 2010: calculating evolutionary conservation in sequence and structure of proteins and nucleic acids. *Nucleic Acids Res.* **38**, W529-533 (2010).

S3. Chung, J., *et al.* Small-molecule inhibitor binding to an N-acyl-homoserine lactone synthase. *Proc. Natl. Acad. Sci. U. S. A.* **108**, 12089-12094 (2011).

S4. Soding, J., Biegert, A., & Lupas, A. N. The HHpred interactive server for protein homology detection and structure prediction. *Nucleic Acids Res.* **33**, W244-248 (2005).

S5. Gould, T. A., Schweizer, H. P. & Churchill, M. E. Structure of the *Pseudomonas aeruginosa* acyl-homoserinelactone synthase LasI. *Mol. Microbiol.* **53**, 1135-1146 (2004).

S6. Watson, W. T., Minogue, T. D., Val, D. L., von Bodman, S. B. & Churchill, M. E. Structural basis and specificity of acyl-homoserine lactone signal production in bacterial quorum sensing. *Mol. Cell* **9**, 685-694 (2002).

S7. Van Wagoner, R. M. & Clardy, J. FeeM, an N-acyl amino acid synthase from an uncultured soil microbe: structure, mechanism, and acyl carrier protein binding. *Structure* **14**, 1425-1335 (2006).

S8. Siehl, D. L., Castle, L. A., Gorton, R. & Keenan, R. J. The molecular basis of glyphosate resistance by an optimized microbial acetyltransferase. *J. Biol. Chem.* **282**, 11446-11455 (2007).

S9. Yachdav, G., *et al*. PredictProtein--an open resource for online prediction of protein structural and functional features. *Nucleic Acids Res.* **42**, W337-343 (2014).

S10. Eswar, N., *et al*. Comparative protein structure modeling using Modeller. *Curr. Protoc. Bioinformatics Chapter* **5**, Unit 5.6 (2006).

S11. Adams, P. D., *et al.* PHENIX: a comprehensive Python-based system for macromolecular structure solution. *Acta Crystallogr. D Biol. Crystallogr.* **66**, 213-221 (2010).

S12. Laskowski, R. A. PDBsum: summaries and analyses of PDB structures. *Nucleic Acids Res.* **29**, 221-222 (2001).

S13. Benkert, P., Kunzli, M., & Schwede, T. QMEAN server for protein model quality estimation. *Nucleic Acids Res.* **37**, W510-514 (2009).

S14. Robert, X., & Gouet, P. Deciphering key features in protein structures with the new ENDscript server. *Nucleic Acids Res.* **42**, W320-324 (2014).

S15. The PyMOL Molecular Graphics System, Version 1.8 Schrödinger, LLC. www.pymol.org

S16. Schuttelkopf, A. W. & van Aalten, D. M. PRODRG: a tool for high-throughput crystallography of protein-ligand complexes. *Acta Crystallogr. D Biol. Crystallogr.* **60**, 1355-1363 (2004).

S17. Grosdidier, A., Zoete, V. & Michielin, O. SwissDock, a protein-small molecule docking web service based on EADock DSS. *Nucleic Acids Res.* **39**, W270-277 (2011).

S18. Madadkar-Sobhani, A. & Guallar, V. PELE web server: atomistic study of biomolecular systems at your fingertips. *Nucleic Acids Res.* **41**, W322-328 (2013).

S19. Borrelli, K. W., Vitalis, A., Alcantara, R. & Guallar, V. PELE: Protein Energy Landscape Exploration. A Novel Monte Carlo Based Technique. *J. Chem. Theory Comput.* **1**, 1304-1311 (2005).

S20. Deshong, P., Li, W., Kennington, J. W., Ammon, H. L. & Leginus, J. M. M. A nitrone-based cycloaddition approach to the synthesis of the glycosyl system of nogalomycin, menogaril, and their congeners. *J. Org. Chem.* **56**, 1364-1373 (1991).

S21. Cronan, J. E. & Thomas, J. Bacterial fatty acid synthesis and its relationships with polyketide synthetic pathways. *Methods Enzymol.* **459**, 395-433 (2009).

S22. Martin, A. *et al*. A new rapid and simple colorimetric method to detect pyrazinamide resistance in *Mycobacterium tuberculosis* using nicotinamide. *J. Antimicrob. Chemother.* **58**, 327-331 (2006).
